# PRMT5 functionally associates with EZH2 to promote colorectal cancer progression through epigenetically repressing CDKN2B expression

**DOI:** 10.7150/thno.53023

**Published:** 2021-01-27

**Authors:** Liu Yang, Da-wei Ma, Yue-peng Cao, Dong-zheng Li, Xin Zhou, Ji-feng Feng, Jun Bao

**Affiliations:** 1 Department of Colorectal Surgery, The Affiliated Cancer Hospital of Nanjing Medical University & Jiangsu Cancer Hospital & Jiangsu Institute of Cancer Research, Nanjing, China.; 2 Department of Pathology, The Affiliated Cancer Hospital of Nanjing Medical University & Jiangsu Cancer Hospital & Jiangsu Institute of Cancer Research, Nanjing, China.; 3 Department of Chemotherapy, The Affiliated Cancer Hospital of Nanjing Medical University & Jiangsu Cancer Hospital & Jiangsu Institute of Cancer Research, Nanjing, China.

**Keywords:** PRMT5, Proliferation, CDKN2B, EZH2, CRC

## Abstract

**Background:** Protein arginine methyltransferase 5 (PRMT5) is a type II arginine methyltransferase that symmetrically di-methylates arginine residues on both histone and non-histone protein substrates. Accumulating evidence suggests that PRMT5 exerts its oncogenic properties in a wide spectrum of human malignancies. However, the underlying mechanisms by which PRMT5 contributes to the progression of colorectal cancer (CRC) remain to be defined.

**Methods:** Western blot and real-time PCR were used to analyze the expression of CDKN2B. Co-immunoprecipitation (Co-IP), immunofluorescence and GST pulldown assays were employed to investigate the interaction between PRMT5 and EZH2. Luciferase reporter and chromatin immunoprecipitation (ChIP) assays were performed to validate CDKN2B as a direct target of PRMT5/EZH2. DNA methylation status at the CpG islands of promoter region of CDKN2B gene was analyzed by bisulfite sequencing. The effect of PRMT5/EZH2 on malignant phenotypes was examined through *in vitro* and *in vivo* assays. PRMT5 and EZH2 protein expression levels in CRC tissues were analyzed by immunohistochemistry (IHC) staining.

**Results:** We observed that PRMT5-deficient CRC cells exhibit proliferation defects* in vitro*. PRMT5 was identified as a major transcriptional repressor of CDKN2B (p15^INK4b^) for determining CRC progression. Mechanistically, PRMT5-mediated histone marks H4R3me2s and H3R8me2s were predominantly deposited at the promoter region of CDKN2B gene in CRC cells. Knockdown of PRMT5 in CRC cells decreased the accumulation of H4R3me2s and H3R8me2s marks and reduced the CpG methylation level of CDKN2B promoter, then re-activated CDKN2B expression. Strikingly, silencing of CDKN2B partially abrogated the proliferation defects caused by PRMT5 depletion *in vitro* and *in vivo*. Furthermore, we proved that PRMT5 interacted with Enhancer of zeste homolog 2 (EZH2), leading to enhanced EZH2 binding and H3K27me3 deposition together with decreased transcriptional output of CDKN2B gene. Importantly, we found that the combined interventions exerted a synergistic inhibitory effect of combined treatment with PRMT5i (GSK591) and EZH2i (GSK126) on the growth of CRC cells/xenografts* in vitro* and *in vivo*. Moreover, PRMT5 and EZH2 were found to be significantly elevated and associated with poor prognosis in CRC patients.

**Conclusion:** PRMT5 functionally associates with EZH2 to promote CRC progression through epigenetically repressing CDKN2B expression. Thus, our findings raise the possibility that combinational intervention of PRMT5 and EZH2 may be a promising strategy for CRC therapy.

## Introduction

Colorectal cancer (CRC) is the third most common diagnosed cancer in the world 1. More than 1.4 million new cases are diagnosed with CRC annually, causing nearly 700,000 deaths worldwide 2. Colorectal carcinogenesis is a complex, multistep and multi-genetic process involving an accumulation of multiple genetic and/or epigenetic alterations. The subsequent accumulation of genetic and/or epigenetic alterations, including those activating the proto-oncogenes and inactivating tumor suppressor genes, drive a progression from adenoma to adenocarcinoma and, ultimately, invasive and metastatic adenocarcinoma 3.

Arginine (R) methylation is emerging as an important post-translational modification (PTM) involved in a wide spectrum of intracellular events, including pre-mRNA splicing, gene transcription and mRNA translation 4. Protein arginine methyltransferases (PRMTs) are the enzymes that transfer the methyl group from S-adenosylmethionine (SAM) to the guanidine nitrogen atoms of arginine, resulting in the formation of ω-N^G^-monomethylarginine (MMA), ω-N^G^, N^G^-asymmetric dimethylarginine (ADMA), and ω-N^G^, N^G^-symmetric dimethylarginine (SDMA) 5. Protein arginine methyltransferase 5 (PRMT5), also known as a human homologue of Shk1 kinase-binding protein 1 (SKB1), is a type II arginine methyltransferase that symmetrically di-methylates arginine residues on both histone and non-histone protein substrates 6. Multiple lines of compelling evidence indicate that PRMT5 is implicated in modulation of a wide range of essential cellular events, for example, ribosome biogenesis, cell growth, germ cell development, Golgi apparatus homeostasis and RNA metabolism 7. By now it is widely accepted that PRMT5 exerts oncogene-like activities and its elevation is highly correlated with unfavorable outcomes in patients with lung cancer 8, breast cancer 9, gastric cancer 10, ovarian cancer 11, prostate cancer 12 and glioblastoma 13. Despite a considerable amount of scientific evidence, it is however still scarcely understood how elevated PRMT5 levels promotes tumorigenesis and cancer progression, especially in CRC.

Enhancer of zeste homolog 2 (EZH2) is a catalytic component of Polycomb repressive complex 2 (PRC2) that specially catalyzes tri-methylation of histone H3 at Lys 27 (H3K27me3) by SET domain in its C-terminus 14, 15. Accumulating evidence has shown that EZH2-catalyzed H3K27me3 is implicated in facilitating tumor growth and metastasis through epigenetically repressing its downstream target genes in many human malignancies 16, 17. A vast number of studies suggested that EZH2 is dramatically elevated in a wide spectrum of human malignancies, such as those of the lung 18, breast 19, prostate 20, gastric 21 and hematological malignancies 22, which provides a promising target for anti-cancer drug therapy. In particular, selective inhibition of EZH2 by small-molecular inhibitors or siRNA/shRNA was reported to attenuate cell proliferation *in vitro*, as well as tumor growth *in vivo* 23. The emerging and diverse roles of EZH2 in human cancers mainly derive from its transcriptional, post-transcriptional and post-translational modulations of gene expression 24. However, because EZH2 is usually associated with other epigenetic events in a context-dependent manner, the precise mechanism by which EZH2 modulates the progression of a number of human malignancies remains to be defined.

PRC2 consists of four core subunits, EZH2, SUZ12 (suppressor of zeste 12 homolog), and EED (embryonic ectoderm development), and RBBP4/7 (retinoblastoma binding protein 4/7), and is involved in transcriptional silencing of growth and development-related genes 25, 26. Previous work has shown that BRD7, SUZ12 and PRMT5 co-localize on ST7 and retinoblastoma-like protein 2 (RBL2) promoters, and that their association with these target tumor-suppressor genes correlates with methylation of H3R8, H4R3 and H3K27 in patient-derived mantle cell lymphoma (MCL) and chronic lymphocytic leukemia (CLL) cell lines 27. Functionally, together with methylated CpG islands, the H4R3me2s as well as H3K27me3 marks serves as 'repressive histone marks' which potentially silence a tumor suppressor gene's transcription machinery due to the remodeling of the chromatin 28-30. Given these results and the fact that expression of PRMT5 and EZH2 is significantly elevated in a variety of cancer cells, we reasoned that PRMT5 epigenetically represses gene transcription through association with EZH2 to suppress gene transcription.

In the present study, we report that PRMT5 facilitates the cell proliferation *in vitro* and tumor growth *in vivo*. Mechanistic studies revealed that PRMT5 epigenetically represses the transcription of CDKN2B (p15^INK4b^) gene through association with EZH2 in CRC cells. Further analysis showed that the combined interventions exerted a synergistic inhibitory effect of combined treatment with PRMT5i (GSK591) and EZH2i (GSK126) on the growth of HCT116 cells/xenografts* in vitro* and *in vivo*. Our data support the concept that combinational inhibition of PRMT5 and EZH2 may be a promising strategy for CRC therapy.

## Materials and Methods

### Cell culture

Human CRC cell lines (HCT116 and SW480) and human embryonic kidney cell line (HEK-293T) were obtained from the Cell Bank of the Chinese Academy of Sciences (Shanghai, China). HCT116 (poorly differentiated) and NCM460 (noncancerous colon) cells were culture in McCoy's 5A medium (Modified, Gibco). SW480 (moderately differentiated) and HEK-293T cells were cultured in Dulbecco's modified Eagle's medium (DMEM, Gibco). All culture media were supplemented with 10% fetal bovine serum (FBS, Gibco) and 1% antibiotics (Invitrogen; 100 U/ml penicillin and 100 mg/mL streptomycin). CRC cells were incubated at 37 °C in a humidified incubator with an atmosphere of 5% CO_2_.

### Lentivirus-mediated gene silencing

Short hairpin RNAs (shRNAs) as well as a control shRNA were designed to silence PRMT5, EZH2 or CDKN2B. Control shRNA: 5'-TTCTCCGAACGTGT CACGT-3'; shPRMT5-1: 5'-GGGACTGGAATACGCTAATTG-3'; shPRMT5-2: 5'-GGAATCTCAGACATATGAAGT-3'; shEZH2-1: 5'-GCTAGGTTAATTGGGACCA AA-3'; shEZH2-2: 5'-CCAACACAAGTCATCCCATTA-3'; shCDKN2B: 5'-GGTGC GACAGCTCCTGGAA-3'. DNA sequences coding corresponding shRNAs were synthesized and then inserted into the lentiviral plasmid pLKO.1-TRC. psPAX2 (packaging plasmid) and pMD2.G (enveloping plasmid) and pLKO.1-TRC-shRNA were transiently transfected into HEK-293T cells using Lipofectamin 2000 (Life Technologies) to prepare the lentiviruses. 48 ~ 72 hours later after transfection, culture supernatants were harvested and filtered through 0.22 μm-pore-sized filters (Millipore, C8848). For virus infection, HCT116, SW480 or NCM460 cells were treated with harvested viral supernatants. Polybrene (5 μg/mL; Sigma-Aldrich) was added to the cultures to improve the infection efficiency as an enhancing reagent. 48 ~ 72 hours later, infected HCT116, SW480 or NCM460 cells were selected with puromycin (1 μg/mL; Sigma-Aldrich) for 5 ~ 7 days.

### Evaluation of proliferative activity

A CCK-8 Cell Counting Kit (Beyotime, C0037) was used to assess the proliferation of HCT116 and SW480 cells. Cells were seeded in 96-well plates at a density of 2×10^4^ cells/mL (100 µL/well) and incubated at 37 °C with 5% CO_2_. The optical density of the solution (OD450) in each well was measured by spectrophotometry (BioTek) on days 0, 1, 2 and 3 for HCT116 and SW480 cell, following addition of 10 µL CCK-8 reagent.

For EdU (5-ethynyl-2'-deoxyuridine) assay, cells were seeded in 96-well plates at a density of 5 × 10^4^ cells per well in a 24-well plates and incubated at 37 °C with 5% CO_2_ overnight. The EdU incorporation assay was carried out according to the instructions for the BeyoClick™ EdU-488 assay kit (Beyotime, C0071S). After incubation with 10 μM EdU for 2 h, cells were fixed with 4% paraformaldehyde, permeabilized with 0.3% Triton X-100, and stained with fluorescent dyes (green). 1 × Hoechst 33342 was used to stain the cell nuclei (blue; 1:1000) at room temperature for 10 min. Cells were observed under a fluorescent microscope (Nikon).

### Colony-formation assay

Plate colony-formation assay was performed to determine the colony-formation capabilities of HCT116 and SW480 cells. Cells were seeded at 1×10^3^ cells per well in 6-well plates and then cultured in medium supplemented with 10% FBS and 1% antibiotics (100 U/mL penicillin and 100 mg/mL streptomycin) at 37 °C with 5% CO_2_. After macro-clones (>50 cells/colony) formed, cells were fixed with 100% methanol and stained with 0.1% (m/v) crystal violet solution at room temperature.

### RNA extraction, reverse transcription and real-time PCR

Total RNA was extracted using Trizol reagent (Beyotime, R0016) and the ratio of A260/A280 was examined by a NanoDrop 2000 spectrophotometer (Thermo Scientific). A total of 0.5 ~ 1 µg of purified RNA was reversed-transcribed to cDNA with the BeyoRT™ II First Strand cDNA Synthesis Kit with gDNA Eraser (Beyotime, D7170S) in the following conditions: 65 °C for 5 min, 42 °C for 60 min and 80 °C for 10 min, and then subjected to real-time PCR analysis. Real-time PCR was performed using the BeyoFast™ SYBR Green qPCR Mix (Beyotime, D7260) on an ABI-7900 instrument (Applied Biosystems). The real-time PCR program steps were as follows: 95 °C for 5 min, then 40 cycles of 95 °C for 30 s, 60 °C for 30 s and 72 °C for 30 s. The 2^-ΔΔCt^ method was used to calculate relative mRNA amounts of target genes to the endogenous GAPDH control. The sequences of real-time PCR primers were listed in Table S1.

### Protein extraction and immunoblotting

Whole-cell lysates of HCT116 and SW480 cells were extracted using lysis buffer, including 50 mM Tris-HCl (pH 7.4), 150 mM NaCl, 1 mM EDTA, 1% Triton X-100, 0.1% SDS, 1% sodium deoxycholate and 1 mM phenylmethylsulfonyl fluoride (PMSF). Cell lysates were centrifuged at a speed of 14000 rpm for 15 min, then mixed with 5×loading buffer and denatured at 100 °C for 5 min. Aliquots of supernatant protein (40 ~ 50 μg) were separated by sodium dodecyl sulfate-polyacrylamide gel electrophoresis (SDS-PAGE) and electrophoretically transferred to a polyvinylidene fluoride (PVDF) membrane (Millipore). The PVDF membranes were then blocked for 1 h with 5% (w/v) non-fat milk at room temperature. After incubation with anti-PRMT5 (Abcam, ab109451), anti-EZH2 (ProteinTech, 21800-1-AP), anti-CDKN2B (Abcam, ab53034) or anti-GAPDH (ProteinTech, 60004) overnight at 4°C, the PVDF membranes were washed with PBST and incubated with corresponding horseradish peroxidase (HRP)-conjugated secondary antibody (Sigma-Aldrich) at room temperature. Subsequently, the immunoreactive bands were visualized using ECL detection reagent (Thermo Scientific).

### Recombinant protein expression and GST pulldown

Glutathione S-transferase (GST) recombinant proteins were expressed in *E. coli* strain BL21 (DE3) transformed with pGEX plasmids encoding GST or GST-PRMT5 fusion protein. Briefly, the plasmids were transformed into *E. coli* strain BL21 (DE3), and then cultured and induced with 1 mM isopropyl-beta-D-thiogalactopyranoside (IPTG) to express GST or GST-PRMT5 fusion proteins. The GST recombinant proteins were purified with glutathione Sepharose 4B beads (Amersham Pharmacia Biotech) from the lysate of *E. coli* strain BL21 (DE3). Similarly, His-tagged EZH2 fusion protein was expressed in *E. coli* strain BL21 (DE3) and the His-tagged EZH2 fusion protein was purified with Ni-NTA agarose beads (Qiagen). GST-tagged PRMT5 or His-tagged EZH2 recombinant proteins were analyzed by sodium dodecyl sulfate-polyacrylamide gel electrophoresis (SDS-PAGE) and stained with Coomassie blue to visualize His or GST fusion proteins. Subsequently, GST pulldown assays were performed to validate the direct interaction of GST-tagged PRMT5 and His-tagged EZH2 *in vitro*. Equal amount of GST or GST-tagged PRMT5 fusion proteins were incubated with His-tagged EZH2. After washing, the bound fusion proteins were eluted from glutathione Sepharose 4B beads, separated by SDS-PAGE and detected by immunoblotting with anti-EZH2 antibody.

### Luciferase reporter gene assays

Luciferase reporter gene assays were used to monitor the transcriptional activity of specific genes *in vitro*. To obtain promoter-luciferase reporter constructs, promoter region of the human CDKN2B gene (-2000 to -1 bp) was PCR-amplified from genomic DNA, cloned into the upstream of the luciferase reporter gene in the promoter-less pGL3.0-empty vector (Promega) and confirmed by DNA sequencing. Next, these constructs and PRMT5-overexpressing plasmid (pCMV-PRMT5) were co-transfected into HCT116 and SW480 cells using Lipofectamin 2000 (Life Technologies). Transiently transfected HCT116 and SW480 cells were cultured for 48 ~ 72 hours and then lysed with lysis buffer (Beyotime, P0013). The luciferase activity was assessed using the dual luciferase reporter assay system (Promega) according to the manufacturer's protocol.

### DNA methylation analysis by bisulfite sequencing

Cells were washed with 1 × PBS, and then the genomic DNA was extracted from the cells using the DNeasy Tissue Kit (Qiagen). DNA methylation status at the CpG islands of promoter region of CDKN2B gene was analyzed by bisulfite sequencing (BS). Bisulfite conversion of genomic DNA was performed using the EZ DNA Methylation-Gold kit (Zymo Research) according to manufacturer's instructions. The bisulfite-treated genomic DNA were subjected to PCR for the amplification of the CpG islands of CDKN2B promoter using Hot Start Takara Taq DNA Polymerase (Takara). Amplified PCR products were cloned into the pGEMT-easy vector (Promega) using DNA Ligation Kit (Takara). Ten insert-positive clones were isolated by the QIAprep Spin Miniprep kit (Qiagen) and sequenced using the ABI sequencing system (Applied Biosystems). The PCR primers used for bisulfite sequencing of CpG islands of promoter region of CDKN2B gene are presented in Table S2.

### Immunofluorescence

Cells were fixed with phosphate-buffered saline (PBS) containing 4% paraformaldehyde for 30 min and permeabilized with Triton X-100 for 40 min at room temperature. Primary antibodies for staining PRMT5 (Sigma, P0493) and EZH2 (ProteinTech, 21800-1-AP) were used at 1:200 and 1:500 for 1 h at room temperature, respectively. After washing with HEPES buffered saline (HBS: 20 mM HEPES, 145 mM NaCl, pH 7.4), cells were incubated with secondary antibodies (Alexa Fluor-488 anti-mouse and Alexa Fluor-546 anti-rabbit) for 40 min. After washing with PBS, cells were stained with 4, 6-diamidino-2-phenylindole dihydrochloride (DAPI; a DNA-specific fluorescent dye) for 5 ~ 10 min. Stained cells were mounted with anti-fade mounting medium (ProLong Gold Antifade), covered with coverslips and visualized with epifluorescence microscope (Nikon).

### Data acquisition from the Oncomine database

The available data was assessed using the Oncomine database (https://www.oncomine.org) according to the following criteria: (I) gene: PRMT5, EZH2 and CDKN2B; (II) cancer type: colorectal cancer; (III) data type: all; (IV) analysis type: cancer analysis vs. normal analysis; (V) thresholds: *P* value <1E-4, fold change >2 and gene rank = top 10%.

### Specimens and immunohistochemistry (IHC) staining

This study was approved by the Ethics Committee of the The Affiliated Cancer Hospital of Nanjing Medical University, while informed consents were provided by all the patients. Surgically derived CRC biopsies were fixed in formalin solutions (10%; v/v), embedded in paraffin and sectioned at 3 ~ 5 μm thickness. The slides were washed with 3% hydrogen peroxide (H_2_O_2_; v/v) to block endogenous peroxidase activity and to avoid immunoreaction. Subsequently, the slides were incubated with the primary antibodies more than 4 hours, including PRMT5 (Sigma, P0493), EZH2 (ProteinTech, 21800-1-AP) and CDKN2B (Abcam, ab53034). After washing with PBS, the sections were incubated with appropriate secondary antibodies conjugated with horseradish peroxidase (HRP; Sigma-Aldrich) and 3, 3'-diaminobenzidine (DAB; Pierce) Substrate solution to visualize the immunoreaction. The staining of CRC slides were reviewed by two trained pathologists blindly and independently. Semi-quantitative of protein expression was calculated according to staining intensity and percentage of positive cells to generate a histological score (H score; range: 0 ~ 300). H score = ΣPi (i + 1), where is the intensity of staining (0 ~ 3) and Pi is the percentage of staining positive cells (0% ~ 100%).

### Chromatin immunoprecipitation (ChIP) assays

To prepare chromatin, cells were collected and cross-linked with 1% formaldehyde for 10 min at room temperature. The crosslinking reaction was stopped by adding glycine (2 M) and the chromatin was sonicated to generate 200 ~ 500 bp DNA fragments. Immunoprecipitation was performed by adding either anti-PRMT5 (Sigma, P0493), anti-H4R3me2s (Abcam, ab5823), anti-H3R8me2s (Abcam, ab272149), anti-EZH2 (Abcam, ab191250), H3K27me3 (Abcam, ab8898), anti-IgG (Abcam, ab172730) and Protein G agarose beads (salmon sperm DNA pre-treated) to each sample and incubated overnight at 4 °C. Following extensive washing with, the complexes were decrosslinked at 65 °C and treated with Proteinase K (Roche). The eluted DNA was precipitated with ethanol at -20 °C in the presence of Glycogen (20 mg/mL, Beyotime, D0812) as a carrier, washed twice with 70% ethanol, and then subjected to real-time PCR using primers specific to the target gene promoters, which were listed in Table S3.

### Combination treatment and index for synergy *in vitro*

The combination therapies were performed in 96-well plates. Cells were treated with PRMT5 inhibitor-GSK591 (MCE, HY-100235), EZH2 inhibitor-GSK126 (MCE, HY-13470), or the combination at graded concentrations for 7 days, and cell proliferation was determined by CCK-8 assays. To determine the presence of a possible synergistic effect of PRMT5i (GSK591) and EZH2i (GSK126), the combination index (CI) was calculated using CompuSyn software. A CI < 0.8 indicates synergy and CI > 1.0 indicates an antagonistic effect.

### Animal experiments

Six-week-old female nude mice (BALB/c nu/nu; 20 g body weight) used for the *in vivo* experiments were purchased from the Experimental Animal Center, Chinese Academy of Sciences (Shanghai, China). All the nude mice were housed under a standardized light-dark cycle (12:12) in standard cages and allowed to free access to food and water. The experimental protocol and all animal experimental procedures were approved by the Animal Care and Use Committee of the Affiliated Cancer Hospital of Nanjing Medical University. Cells were suspended in FBS-free McCOY's 5A medium at a concentration of 2×10^7^ cells/mL, and then implanted subcutaneously into the mouse right flank of each mouse (2×10^6^ cells per mouse). The mice were followed for the observation of xenograft growth and measured by manual caliper every three days.

For *in vivo* drug studies, tumor-bearing mice were randomized into four groups (7 mice per group), and drug dosing was initiated when average tumor volume reached 50 mm^3^ (at day 4). PRMT5i (GSK591; MCE, HY-100235) and EZH2i (GSK126; MCE, HY-13470) were dissolved in 20% Sulfobutylether-β-Cyclodextrin (SBE-β-CD, pH 4 ~ 4.5). GSK591 and GSK126 were administered intraperitoneally every other day at a dose of 100 mg/kg for a month. Tumor growth was monitored by the measurement of subcutaneous tumor volume as above. Mouse body weight was also measured by manual caliper every four days. The tumor volumes were calculated as 0.5 × length × width^2^. At the end point, mice were sacrificed, and xenografts were surgically isolated, weighted and photographed. Tumor growth inhibition (TGI) rate was calculated as shown: TGI = [1 - (tumor volume of control on day 36 - tumor volume of treated on day 4) / (tumor volume of control on day 36 - tumor volume of control on day 4)] × 100.

### Statistical analysis

All statistical analysis was performed using GraphPad Prism 7.0 (GraphPad Software, Inc., California, USA). Statistical analyses were performed using two-tailed Student's *t*-test to evaluate the significance of the differences between two groups. To analyze the relationship between positive PRMT5/EZH2 expression and clinicopathological parameters of CRC patients, student's *t*-test was used to compare values for the two parameters in each category. Survival was assessed by the Kaplan-Meier method and the survival curves were compared using the log-rank test. Data were reported as the mean ± SD from three independent experiments. *P* values < 0.05 (two-tailed) were considered statistically significant. * and ** are denoted as *P* < 0.05 and *P* < 0.01, respectively.

## Results

### PRMT5 depletion retards CRC cell proliferation *in vitro*

**Figure 1:**
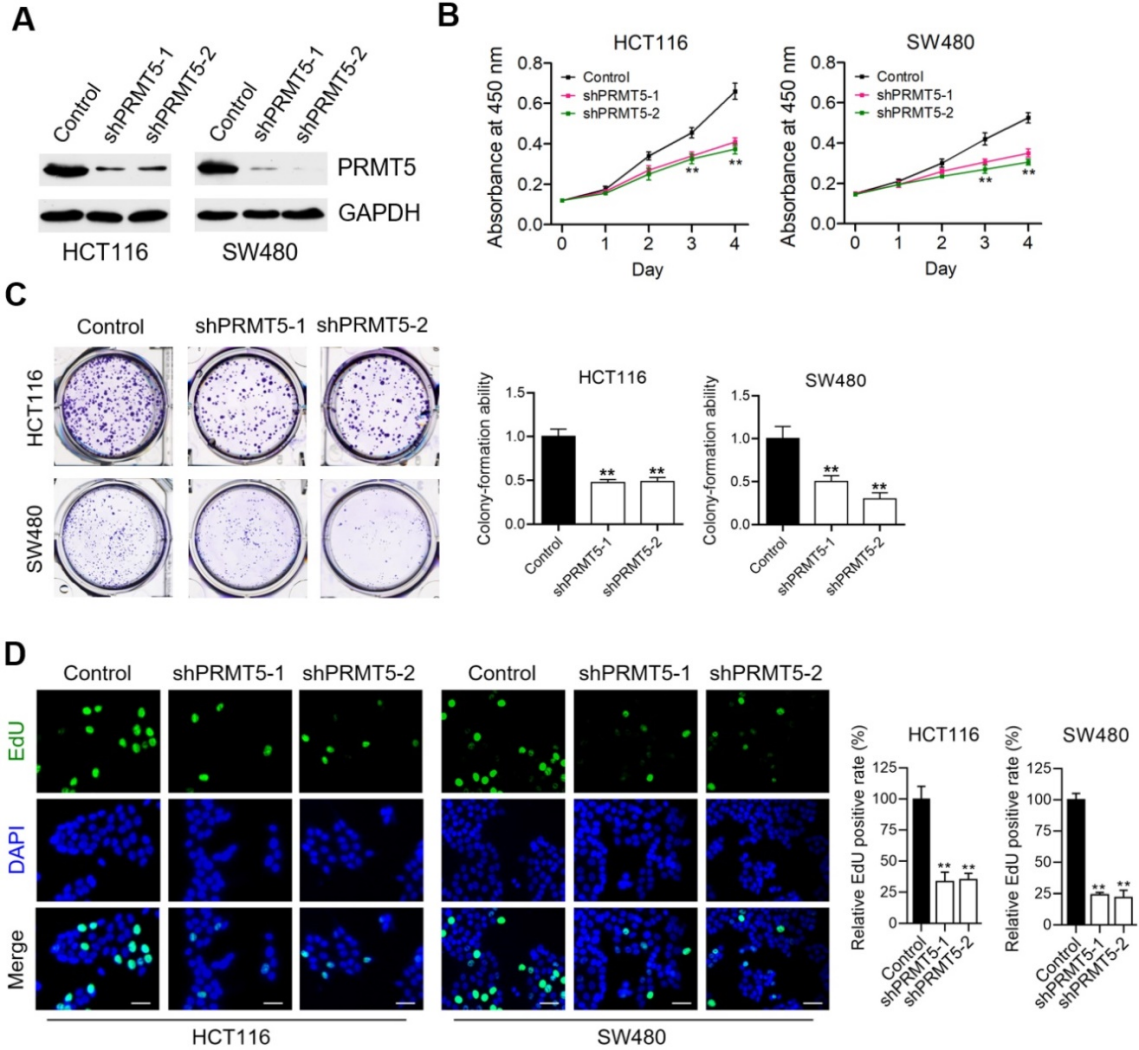
** PRMT5 depletion retards CRC cell proliferation *in vitro*.** (**A**) Immunoblot analysis of PRMT5 expression in control, shPRMT5-1 and shPRMT5-2-infected HCT116 and SW480 cells. GAPDH was used as loading control. (**B**) Cell proliferation of HCT116 and SW480 cells treated with control, shPRMT5-1 and shPRMT5-2 was evaluated by CCK-8 assay at the same time point of each day. **P < 0.01. (**C**) Colony formation capabilities of HCT116 and SW480 cells stably expressing control shRNA, shPRMT5-1 and shPRMT5-2 were performed by plate colony-formation assays. Quantifications are shown on the right panel. **P < 0.01. (**D**) Representative fluorescence images of control, shPRMT5-1 and shPRMT5-2-treated HCT116 and SW480 cells after EdU incorporation (green). Nuclei are indicated by Hoechst (blue) fluorescence. Quantification of the relative EdU positive rate is shown on the right panel. Scale bar = 20 μm. **P < 0.01.

To understand the contributing roles of PRMT5 on CRC progression, we firstly evaluated the effects of lentivirus-mediated shRNA targeting PRMT5 on cell proliferation of human CRC cell lines-HCT116 and SW480. We observed that the expression levels of PRMT5 in shPRMT5 lentivirus-infected HCT116 and SW480 cells were considerably lower than that of control cells using Western blot (Figure 1A) and immunofluorescence (Figure S1). Cell proliferation analysis measured by CCK-8 assays revealed that knockdown of PRMT5 robustly reduced the proliferative activities of HCT116 and SW480 cells (Figure 1B; **P < 0.01). Marked decreases in colony-forming abilities were also evident in PRMT5-deficient HCT116 and SW480 cells (Figure 1C; **P < 0.01). Ethynyl-2'-deoxyuridine (EdU) incorporation is an effective and precise way to evaluate cell proliferation *in vitro*. As shown in Figure 1D (**P < 0.01), we observed that shRNA-mediated depletion of PRMT5 greatly inhibited EdU incorporation in HCT116 and SW480 cells. These findings together suggest that PRMT5 is associated with a highly proliferative tumor phenotype in CRC cells, emphasizing the critical role of PRMT5 in regulating CRC progression.

### PRMT5 epigenetically regulates CDKN2B expression

**Figure 2:**
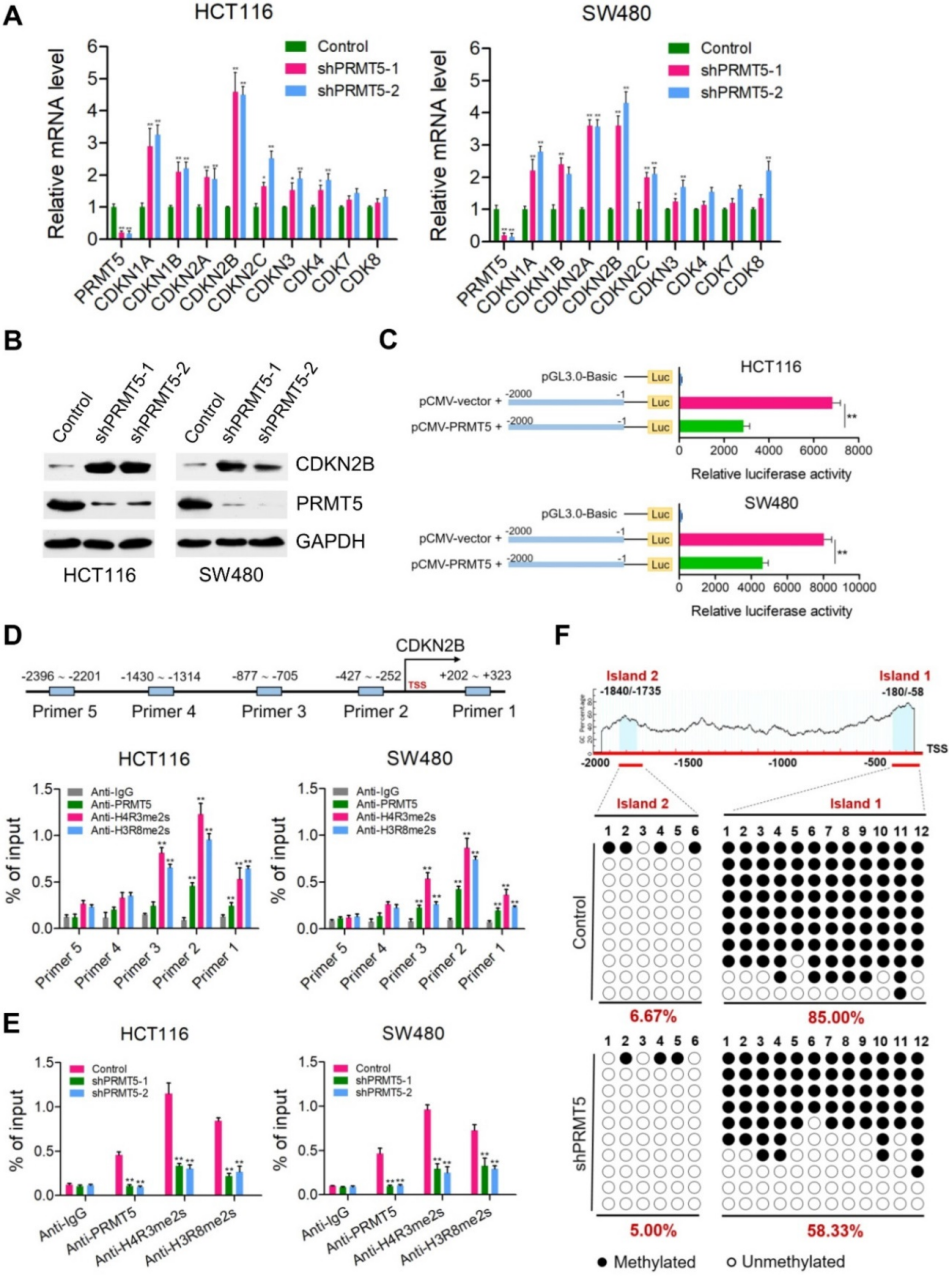
** PRMT5 epigenetically represses CDKN2B expression.** (**A**) Relative mRNA expression of genes involved in the regulation of cell proliferation and cell cycle was determined by real-time PCR in shPRMT5-1 and shPRMT5-2-infected HCT116 and SW480 cells compared with control cells. GAPDH was used as an internal control. *P < 0.05, **P < 0.01. (**B**) Immunoblot analysis of CDKN2B (p15^INK4b^) expression in shPRMT5-1 and shPRMT5-2-infected HCT116 and SW480 cells compared with control cells. GAPDH was used as loading control. (**C**) Promoter-driven luciferase reporter-gene assays showed that the luciferase activity dramatically decreased in HCT116 and SW480 cells transfected with pGL3.0 luciferase reporter plasmid containing promoter fragment of CDKN2B gene (-2000 to -1 bp) and pCMV-PRMT5 vector, compared with that of cells co-transfected with pGL3.0-promoter (-2000 to -1 bp) and pCMV empty vector. **P < 0.01. (**D**) Relative enrichment of PRMT5 and its catalytic histone marks H4R3me2s and H3R8me2s on the promoter region of CDKN2B gene was evaluated by ChIP-qPCR assays in HCT116 and SW480 cells. Primer 1 (+202 ~ +323), Primer 2 (-427 ~ -252), Primer 3 (-877 ~ -705), Primer 4 (-1430 ~ -1314) and Primer 5 (-2396 ~ -2201). IgG was used as a negative control. **P < 0.01. (**E**) Relative enrichment of PRMT5 and its catalytic histone marks H4R3me2s and H3R8me2s on the promoter region of CDKN2B gene (Primer 2: -427 ~ -252) was evaluated by ChIP-qPCR assays in PRMT5-depleted HCT116 and SW480 cells. IgG was used as a negative control. **P < 0.01. (**F**) Bisulfite sequencing analysis of the CDKN2B promoter (CpG island 1: -1840 ~ -1735, CpG island 2: -180 ~ -58) in control and shPRMT5-1-treated HCT116 cells.

Because PRMT5 is a well-established modulator of gene expression, we speculate that one mechanism by which PRMT5 could be modulating CRC progression was through transcriptional control. To investigate this, quantitative real-time PCR analysis was applied to identify downstream transcriptional targets of PRMT5 in HCT116 and SW480 cells. The mRNA expression changes of genes involved in the regulation of cell proliferation and cell cycle were determined, including CDKN1A (p21^CIP1/WAF1^), CDKN1B (p27^KIP1^), CDKN2A (p16^INK4^), CDKN2B (p15^INK4b^), CDKN2C (p18^INK4C^), CDKN3, CDK4, CDK7 and CDK8. We observed that CDKN1A (p21^CIP1/WAF1^), CDKN1B (p27^KIP1^), CDKN2A (p16^INK4^), CDKN2B (p15^INK4b^), CDKN2C (p18^INK4C^) and CDKN3 were significantly elevated when PRMT5 was interfered in both HCT116 and SW480 cells (Figure 2A; *P < 0.05, **P < 0.01), whereas the mRNA expressions of these genes were not significantly changed in noncancerous NCM460 colon cells (Figure S2). Of particular interest, among these candidate target genes, CDKN2B (p15^INK4b^) was the most significantly upregulated target in PRMT5-deficient HCT116 and SW480 cells compared to that in the control cells. Consistent with this result, protein levels of CDKN2B (p15^INK4b^) were also found to be remarkably upregulated in PRMT5-depleted HCT116 and SW480 cells by Western blot (Figure 2B). As expected, we observed that the relative mRNA expression of CDKN2B was significantly downregulated in PRMT5-overexpressed HCT116 and SW480 cells (Figure S3; **P < 0.01), further supporting a potential role of PRMT5 in the transcriptional repression of CDKN2B (p15^INK4b^) expression.

Of particular interest, CDKN2B promoter-driven luciferase reporter-gene assays showed that the luciferase activity dramatically decreased when HCT116 and SW480 cells were co-transfected with pGL3.0-promoter plasmids containing promoter fragment of CDKN2B (-2000 ~ -1 bp) and pCMV-PRMT5 vectors, compared with that of cells co-transfected with pGL3.0-promoter and pCMV-vector (Figure 2C). To further assess the directness of the underlying regulatory mechanism, chromatin immunoprecipitation (ChIP) assays were performed to verify the binding of PRMT5 to CDKN2B promoter. Five pairs of primers were designed across the promoter region of CDKN2B gene. Intriguingly, we observed that PRMT5 was significantly enriched at the core promoter regions of CDKN2B gene in HCT116 and SW480 cells (Figure 2D; **P < 0.01). PRMT5 is known to exert its transcriptional regulatory function by symmetric di-methylation of histone H4 at R3 (H4R3me2s) or histone H3 at R8 (H3R8me2s). Given the fact that PRMT5 substantially attenuated the activity of luciferase reporter gene, it would also be of interest to determine whether PRMT5-deposited H4R3me2s and H3R8me2s, which were identified as transcriptionally repressive histone marks 7, bind to the core promoter region of CDKN2B gene. Mechanistically, ChIP-qPCR assays revealed that the promoter region of CDKN2B gene were predominantly occupied with H4R3me2s and H3R8me2s in HCT116 and SW480 cells. Using independent ChIP-qPCR assays, we observed a significant loss of H4R3me2s and H3R8me2s occupancy at the selected loci (Primer 2) upon PRMT5 depletion (Figure 2E; **P < 0.01). Collectively, our results suggest that CDKN2B (p15^INK4b^) is a direct transcriptional target of PRMT5 in CRC cells.

Previous studies have shown that PRMT5 can elevate the DNA methylation levels of CpG islands located at promoter region of IRX1 or β-globin genes via recruiting DNMT3A 31, 32. These impressive studies prompted us to examine the effects of PRMT5 on the DNA methylation levels of CpG islands located at promoter region of CDKN2B gene. Surprisingly, two potential CpG islands were identified located at the promoter region of CDKN2B (~2.0 kb upstream of the TSS) gene through online MethPrimer software: Island 1: -180 ~ -58 bp and Island 2: -1840 ~ -1735 bp. Bisulfite-sequencing PCR was performed to investigate the methylation status of CpG islands upstream of the TSS in the promoter region of CDKN2B gene. As shown in Figure S4, we observed that CpG Island 1 and 2 located at promoter region of CDKN2B were both hypomethylated in noncancerous NCM460 colon cells (0% and 15%, respectively). Nevertheless, CpG Island 1 was heavily methylated (85.00%), while CpG Island 2 was hypomethylated (6.67%) in HCT116 cells (Figure 2F). Interestingly, we found that methylation levels of CpG Island 1 were remarkably reduced in PRMT5-depleted HCT116 cells (58.33%), compared to that in control cells (85.00%).

### Depletion of CDKN2B expression abrogates the proliferation inhibition caused by PRMT5 knockdown

**Figure 3:**
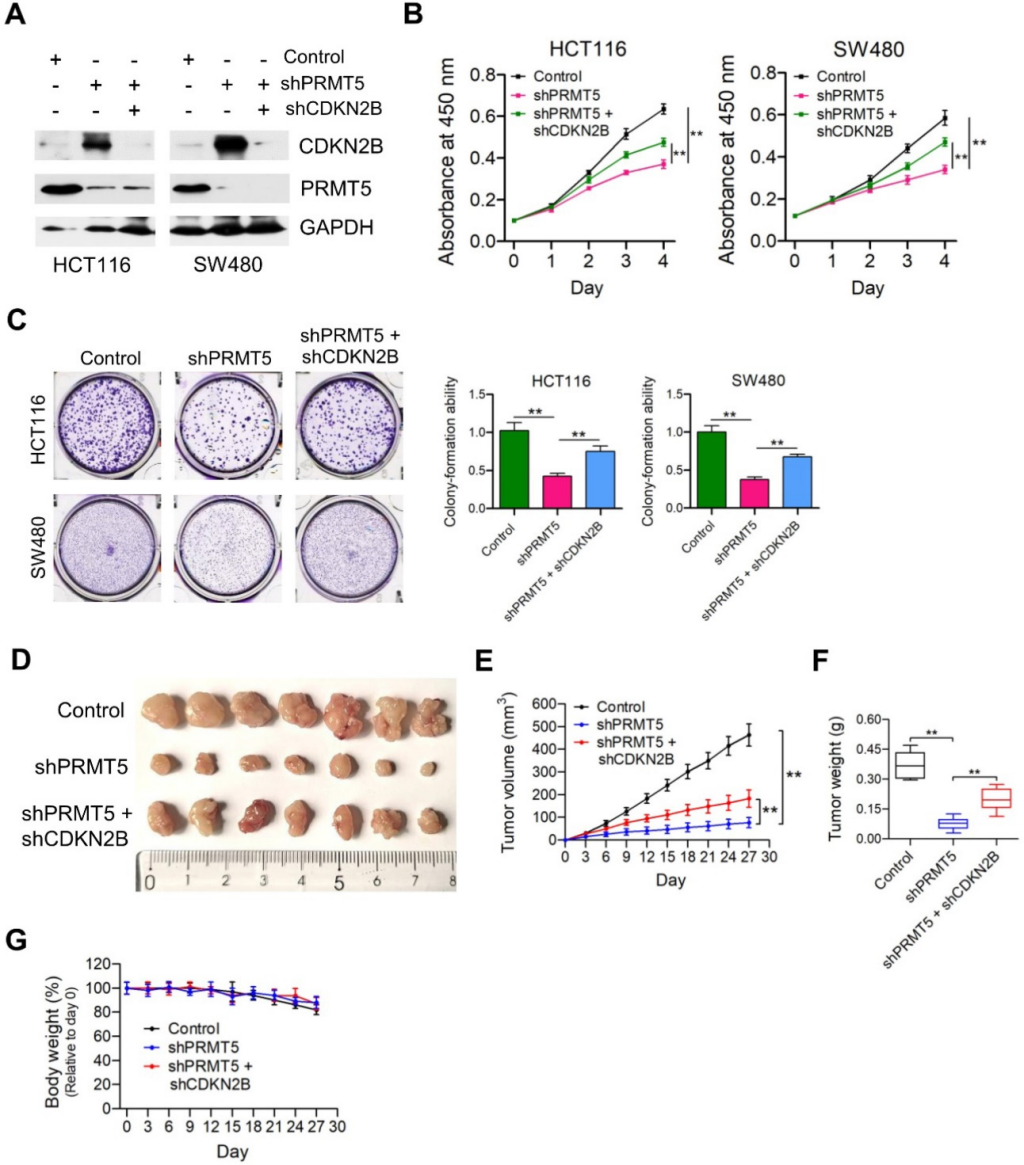
** Depletion of CDKN2B expression abrogates the proliferation inhibition caused by PRMT5 knockdown.** (**A**) Immunoblot analysis of PRMT5 and CDKN2B (p15^INK4b^) expression in HCT116 and SW480 cells treated with control shRNA (negative control), shPRMT5 and shPRMT5 + shCDKN2B. GAPDH was used as loading control. (**B**) Cell proliferation of HCT116 and SW480 cells infected with control shRNA, shPRMT5 and shPRMT5 + shCDKN2B was evaluated by CCK-8 assays at the same time point of each day. **P < 0.01. (**C**) Colony formation capabilities of HCT116 and SW480 cells infected with control shRNA, shPRMT5 and shPRMT5 + shCDKN2B were performed by plate colony-formation assays. Quantifications are shown on the right panel. **P < 0.01. (**D**) Representative images of xenograft tumors excised from mice. Control, shPRMT5 and shPRMT5 + shCDKN2B-infected HCT116 cells were subcutaneously injected into the flank of nude mice. (**E**) Tumor growth curves of control, shPRMT5 and shPRMT5 + shCDKN2B-infected HCT116 xenograft tumors; n = 7. **P < 0.01. (**F**) Tumor weights of control, shPRMT5 and shPRMT5 + shCDKN2B-treated HCT116 xenograft tumors excised from mice. **P < 0.01. (**G**) Body weight (%, relative to day 0) of mice injected with control, shPRMT5 and shPRMT5 + shCDKN2B-treated HCT116 cells. Data are presented as means ± SD from three independent experiments.

Given that PRMT5 has been proposed to contribute to epigenetic control of CDKN2B, we want to determine the effects of CDKN2B on PRMT5 shRNA-mediated growth defects. As shown in Figure 3A, knockdown of CDKN2B expression was verified by Western blot analysis in PRMT5-depleted HCT116 and SW480 cells. Strikingly, we observed that knockdown of CDKN2B partially restored defects in proliferation and colony formation in the PRMT5-depleted HCT116 and SW480 cells, respectively (Figure 3B & C; **P < 0.01). To further support this, we went on to perform *in vivo* experiments with mouse xenograft models. Knockdown of CDKN2B (shPRMT5 + shCDKN2B), at least in part, reversed the growth defect in PRMT5-depleted HCT116 cells (shPRMT5) *in vivo* (Figure 3D-F). Furthermore, there was no significant difference in body weight of the mice among the three groups (Figure 3G). The mRNA expression levels of PRMT5 and CDKN2B in control, shPRMT5 and shPRMT5 + shCDKN2B-treated HCT116 xenograft tumors were verified by real-time PCR (Figure S5). Taken together, the above findings suggest that PRMT5 is required for CRC growth potentially involving CDKN2B gene repression.

### PRMT5 associates with EZH2 in CRC cells

**Figure 4:**
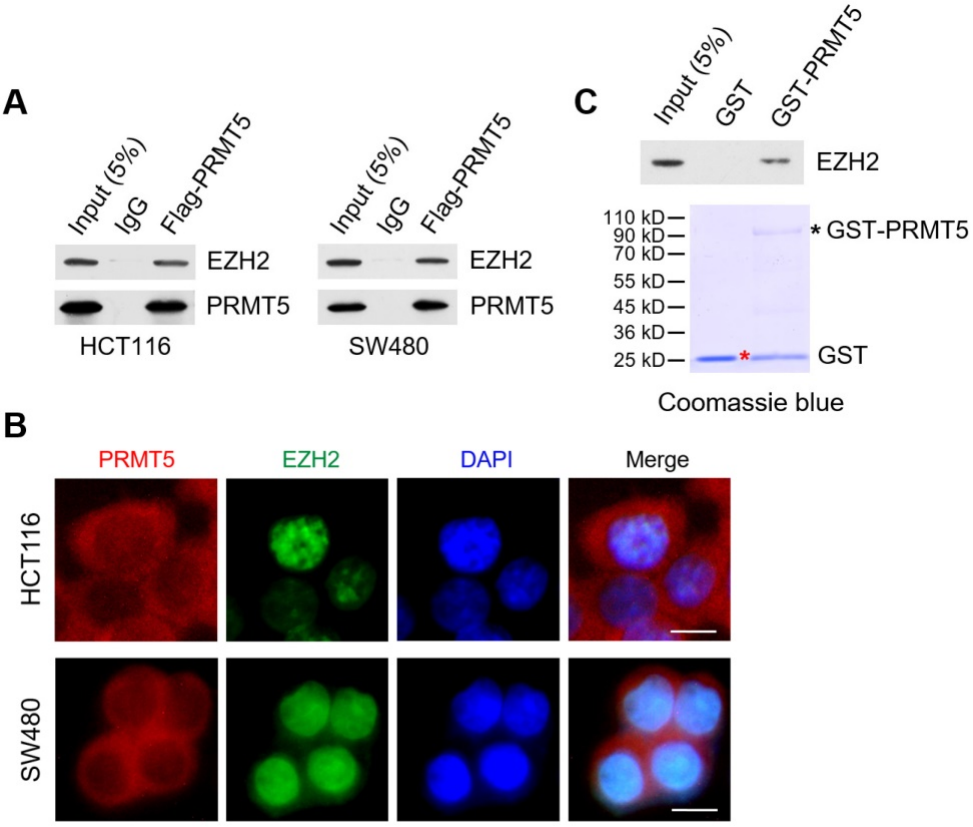
** PRMT5 associates with EZH2 in CRC cells.** (**A**) Co-immunoprecipitation of endogenous EZH2 from HCT116 and SW480 cells overexpressing Flag-tagged PRMT5. IgG was used as the negative control. (**B**) Western blot analysis of EZH2 binding to purified GST and GST-tagged PRMT5 using EZH2 antibody (top). GST and GST-tagged PRMT5 from *E. coli* BL21 (DE3) strain were visualized by staining with Coomassie brilliant blue R-250 (bottom). The red asterisk and black asterisk were GST and GST-tagged PRMT5, respectively. (**C**) The subcellular location of endogenous PRMT5 and EZH2 proteins was analyzed in HCT116 and SW480 cells by immunofluorescence microscopy. Scale bar: 10 µm.

Through reciprocal co-immunoprecipitation (Co-IP) experiments, we show that Flag-tagged PRMT5 interacts with endogenous EZH2 in HCT116 and SW480 cells (Figure 4A). To determine whether PRMT5 and EZH2 co-localized in CRC cells, we performed immunofluorescence staining with anti-PRMT5 and anti-EZH2 antibodies in HCT116 and SW480 cells. In Figure 4B, we observed that endogenous PRMT5 was localized in both the cytoplasm and the nucleus (primarily in the cytoplasm), compared to the predominant presence of EZH2 in the nucleus of HCT116 and SW480 cells. Furthermore, this phenomenon was also found in noncancerous NCM460 colon cells (Figure S6). To establish direct protein-protein interactions *in vitro*, we performed *in vitro* pull-down assays with recombinant Glutathione S‑transferase (GST)-tagged PRMT5, which was expressed in the *E. coli* strain BL21 (DE3) expression system. Strikingly, GST-pulldown assays revealed that GST-tagged PRMT5 (as bait protein) could interact with EZH2 directly, whereas GST alone did not interact with EZH2 (Figure 4C), suggesting a direct interaction of PRMT5 and EZH2 *in vitro*.

### CDKN2B is a direct transcriptional target of EZH2

**Figure 5:**
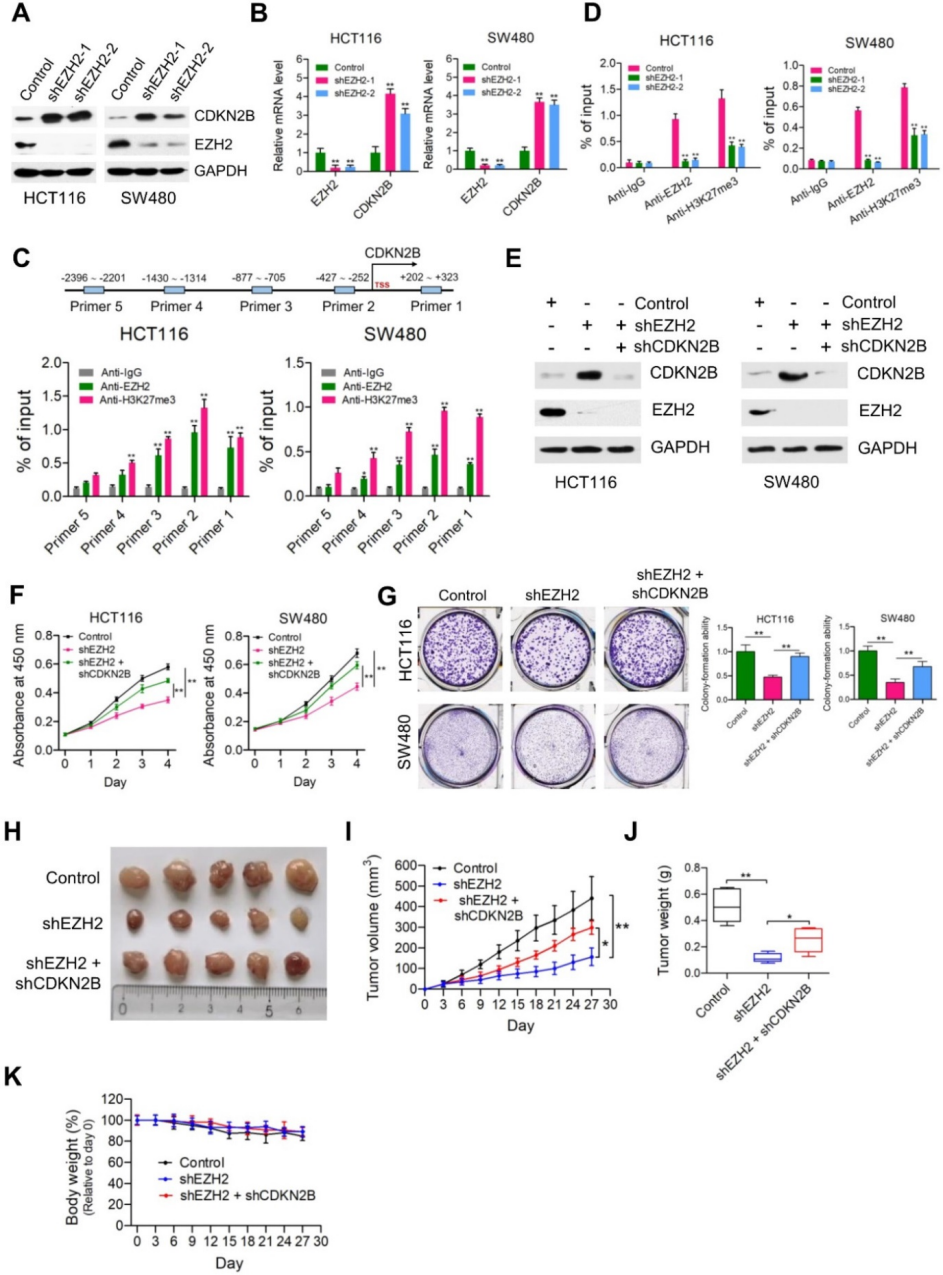
** CDKN2B is a direct transcriptional target of EZH2.** (**A**) Immunoblot analysis of CDKN2B (p15^INK4b^) expression in HCT116 and SW480 cells stably expressing control, shEZH2-1 and shEZH2-2. GAPDH was used as loading control. (**B**) Relative mRNA expression of EZH2 was determined by real-time PCR in HCT116 and SW480 cells stably expressing control shRNA (negative control), shEZH2-1 and shEZH2-2. GAPDH was used as an internal control. **P < 0.01. (**C**) Relative enrichment of EZH2 and its catalytic histone mark H3K27me3 on the promoter region of CDKN2B gene was evaluated by ChIP-qPCR assays in HCT116 and SW480 cells. Primer 1 (+202 ~ +323), Primer 2 (-427 ~ -252), Primer 3 (-877 ~ -705), Primer 4 (-1430 ~ -1314) and Primer 5 (-2396 ~ -2201). IgG was used as a negative control. *P < 0.05, **P < 0.01. (**D**) Relative enrichment of EZH2 and its catalytic histone mark H3K27me3 on the promoter region of CDKN2B gene (Primer 2: -427 ~ -252) was evaluated by ChIP-qPCR assays in EZH2-depleted HCT116 and SW480 cells. IgG was used as a negative control. **P < 0.01. (**E**) Immunoblot analysis of EZH2 and CDKN2B (p15^INK4b^) expression in HCT116 and SW480 cells stably expressing control shRNA, shEZH2 and shEZH2 + shCDKN2B. GAPDH was used as loading control. (**F**) Cell proliferation of HCT116 and SW480 cells stably expressing control, shEZH2 and shEZH2 + shCDKN2B was evaluated by CCK-8 assay at the same time point of each day. **P < 0.01. (**G**) Colony formation capabilities of HCT116 and SW480 cells stably expressing control, shEZH2 and shEZH2 + shCDKN2B were performed by plate colony-formation assays. Quantifications are shown on the right panel. **P < 0.01. (**H**) Representative images of xenograft tumors excised from mice. Control, shEZH2 and shEZH2 + shCDKN2B-treated HCT116 cells were subcutaneously injected into the flank of nude mice. (**I**) Tumor growth curves of control, shEZH2 and shEZH2 + shEZH2-treated HCT116 xenograft tumors; n = 5. *P < 0.05, **P < 0.01. (**J**) Tumor weights of control, shEZH2 and shEZH2 + shCDKN2B-treated HCT116 xenograft tumors excised from mice. *P < 0.05, **P < 0.01. (**K**) Body weight (%, relative to day 0) of mice injected with control, shEZH2 and shEZH2 + shCDKN2B-infected HCT116 cells. Data are presented as means ± SD from three independent experiments.

We next want to determine if EZH2 was directly regulating CDKN2B expression. Real time PCR analysis was performed to determine the protein and mRNA expression levels of CDKN2B (p15^INK4b^) in EZH2-depleted HCT116 and SW480 cells (Figure 5A & B; **P < 0.01). As expected, both protein and mRNA expression levels of CDKN2B (p15^INK4b^) were remarkably elevated in EZH2-deficient HCT116 and SW480 cells compared to the control cells, further supporting a potential role of EZH2 in the transcriptional modulation of CDKN2B (p15^INK4b^) expression. Furthermore, we determined the endogenous expression of EZH2 in NCM460, HCT116 and SW480 cells using Western blot (Figure S7A). We found that the protein expression of EZH2 was noticeably higher in HCT116 and SW480 cells than that in NCM460 cells. Moreover, it seems that PRMT5 depletion has a negligible effect on the transcription of EZH2 in HCT116 and SW480 cells (Figure S7B). Subsequently, ChIP-qPCR assays were performed to verify the binding of EZH2 to the promoter region of CDKN2B gene. We observed that EZH2 was preferentially enriched at the core promoter region of CDKN2B gene in HCT116 and SW480 cells (Figure 5C; *P < 0.05 & **P < 0.01). The polycomb group protein EZH2, which specifically catalyzes the tri-methylation of lysine 27 of histone H3 (H3K27me3), is well established as a transcriptional repressor 15. Therefore, it is reasonable to conclude that EZH2-catalyzed H3K27me3 binds to the core promoter region of CDKN2B gene. Indeed, ChIP-qPCR assays revealed that H3K27me3 was substantially enriched at the core promoter region of CDKN2B gene in HCT116 and SW480 cells. Moreover, independent ChIP-qPCR assays verified the lower occupancy of H3K27me3 at the loci of CDKN2B promoter (Primer 2) in EZH2-deficient HCT116 and SW480 cells (Figure 5D; **P < 0.01). It would also be of interest to determine whether PRMT5 loss affects the depositions of EZH2 and H3K27me3 at the core promoter region of CDKN2B gene. As shown in Figure S8A, ChIP-qPCR assays suggest that PRMT5 knockdown dramatically hampered the enrichments of EZH2 and H3K27me3 at the core promoter region of CDKN2B gene in HCT116 and SW480 cells (*P < 0.05 & **P < 0.01). Meanwhile, the depositions of PRMT5, H4R3me2s and H3R8me2s at the core promoter region of CDKN2B gene were also significantly reduced when EZH2 was silenced in HCT116 and SW480 cells (Figure S8B; **P < 0.01). One possible explanation for this phenomenon is that the interaction of PRMT5 and EZH2 may have a crucial role for maintaining their methyltransferase activities. Altogether, these results indicated that EZH2 and its catalytic histone mark-H3K27me3 were deposited at the core promoter region of CDKN2B gene, strongly demonstrating that CDKN2B (p15^INK4b^) is a direct transcriptional target of EZH2 in CRC cells. Interestingly, we also found that the catalytic marks (H4R3me2s, H3R8me2s and H3K27me3) of PRMT5/EZH2 were also enriched at the core promoter regions of CDKN1A (p21^CIP1/WAF1^), CDKN1B (p27^KIP1^), CDKN2A (p16^INK4^) and CDKN2C (p18^INK4C^) genes (Figure S9; **P < 0.01). These results demonstrated that a cohort of cell cycle-related genes, including CDKN2B (p15^INK4b^), CDKN1A (p21^CIP1/WAF1^), CDKN1B (p27^KIP1^), CDKN2A (p16^INK4^) and CDKN2C (p18^INK4C^) were regulated by PRMT5/EZH2 complexes.

To determine whether EZH2 promotes cell proliferation of CRC cells through repressing CDKN2B expression, we knocked down the expression of CDKN2B in EZH2-deficient HCT116 and SW480 cells (Figure 5E). Similar to PRMT5, knockdown of CDKN2B could significantly restore the defective cell proliferation and colony formation in HCT116 and SW480 cells caused by EZH2 loss (Figure 5F & G; **P < 0.01). Subsequently, *in vivo* experiments with mouse xenograft models showed that knockdown of CDKN2B significantly reversed the growth defect in EZH2-depleted HCT116 xenografts (Figure 5H, I & J), whereas the body weight of mice among these studied groups showed no significant difference (Figure 5K). Moreover, the mRNA expression levels of EZH2 and CDKN2B in control, shEZH2 and shEZH2 + shCDKN2B-treated HCT116 xenograft tumors were verified by real-time PCR (Figure S10; **P < 0.01).

### Synergistic effect of combined treatment with PRMT5i and EZH2i

**Figure 6:**
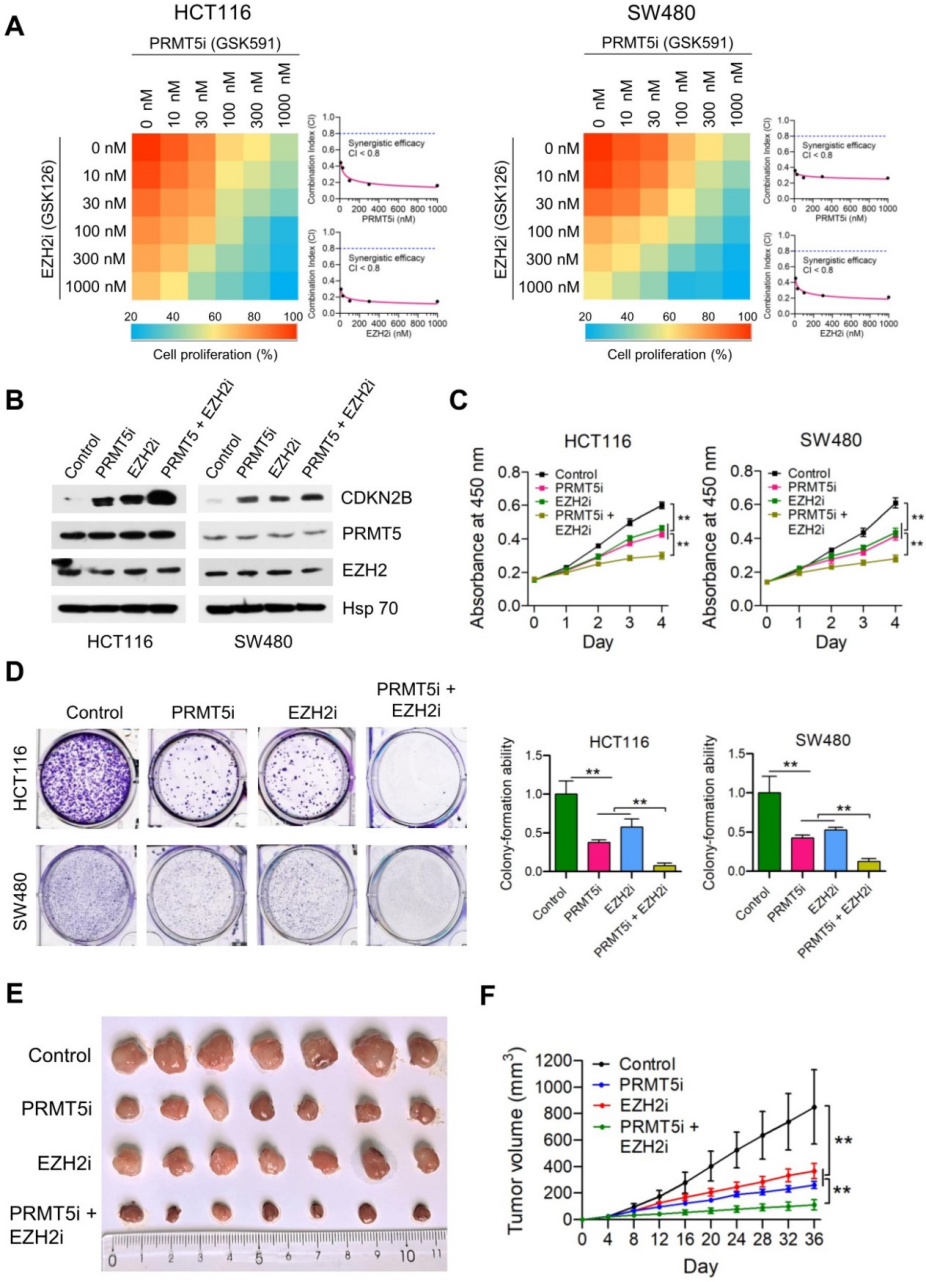
** Synergistic effect of combined treatment with PRMT5i and EZH2i. (A)** Drug dose-response matrix for proliferation inhibition of HCT116 and SW480 cells treated with PRMT5i (GSK591) and EZH2i (GSK126). Color gradation indicates percentage viability at the indicated dose combination. Combination index (CI) plots for 100 nM PRMT5i (GSK591) (top) or EZH2i (GSK126) (bottom) with graded doses of EZH2i (GSK126) or PRMT5i (GSK591) in HCT116 and SW480 cells. (**B**) Immunoblots of PRMT5, EZH2 and CDKN2B (p15^INK4b^) protein levels in HCT116 and SW480 cells following treatment with PRMT5i (GSK591; 100 nM), EZH2i (GSK126; 100 nM), or PRMT5i (GSK591; 100 nM) + EZH2i (GSK126; 100 nM). GAPDH was used as a loading control. (**C**) Cell proliferation of HCT116 and SW480 cells treated with PRMT5i (GSK591; 100 nM), EZH2i (GSK126; 100 nM), or PRMT5i (GSK591; 100 nM) + EZH2i (GSK126; 100 nM) by CCK-8 assays at the same time point of each day. **P < 0.01. (**D**) Colony formation capabilities of HCT116 and SW480 cells treated with PRMT5i (GSK591; 100 nM), EZH2i (GSK126; 100 nM), or PRMT5i (GSK591; 100 nM) + EZH2i (GSK126; 100 nM) by plate colony-formation assays. Quantifications are shown on the right panel. **P < 0.01. (**E**) Representative images of xenograft tumors excised from mice. Control, PRMT5i (GSK591), EZH2i (GSK126), or PRMT5i (GSK591) + EZH2i (GSK126)-treated HCT116 cells were subcutaneously injected into the flank of nude mice. (**F**) Tumor growth curves of Control, PRMT5i (GSK591), EZH2i (GSK126), or PRMT5i (GSK591) + EZH2i (GSK126)-treated HCT116 xenografts; n = 7, **P < 0.01.

Given that PRMT5/EZH2 complex promotes CRC cell proliferation through epigenetic repression of CDKN2B, we sought to test whether combined inhibition of PRMT5 and EZH2 would exert a better therapeutic effect in CRC cells. To this end, we initially examined the effect of a combination of PRMT5i (PRMT5 inhibitor; GSK591) and EZH2i (EZH2 inhibitor; GSK126) on the proliferation of CRC cells. Heatmap and combination index (CI) plots of combination treatment of PRMT5i (GSK591) with EZH2i (GSK126) showed that the two inhibitors synergistically improved the anti-proliferative effect on HCT116 and SW480 cells (CI < 0.8; Figure 6A). Based on these results, designated concentrations of PRMT5i (GSK591; 100 nM) and EZH2i (GSK126; 100 nM) were used in further *in vitro* studies. Western blot analysis clearly showed that the selected combination of PRMT5i (GSK591) and EZH2i (GSK126) robustly activated the protein expression of CDKN2B (Figure 6B). Combinational treatment with PRMT5i (GSK591) and EZH2i (GSK126) dramatically attenuated the cell proliferation and colony formation capabilities of HCT116 and SW480 cells compared to those of the cells treated with PRMT5i (GSK591) or EZH2i (GSK126) alone (Figure 6C & D). Interestingly, we observed that shRNA-mediated CDKN2B knockdown could restore the reduced colony-formation ability of PRMT5i/EZH2i-treated SW480 cells (Figure S11; **P < 0.01).

We also established HCT116-derived xenograft models to test the synergistic benefits of combined treatment with PRMT5i and EZH2i *in vivo*. As shown in Figure 6E & F, single treatment with PRMT5i (GSK591; 2.5 mg/kg) or EZH2i (GSK126; 2.5 mg/kg) had a moderate inhibitory effect (TGI rate: 71.18% and 57.85%, respectively), whereas combined treatment with PRMT5i and EZH2i (GSK591 + GSK126) significantly inhibited tumor growth (**P < 0.01; TGI rate: 88.30%). The mRNA expression levels of PRMT5, EZH2 and CDKN2B in HCT116 xenograft tumors treated with PRMT5i, EZH2i or combined treatment with PRMT5i and EZH2i were verified by real-time PCR (Figure S12). These results indicate that the combined intervention had a synergistic inhibitory effect of combined treatment with PRMT5i and EZH2i on the growth of HCT116 xenografts *in vivo*.

### PRMT5 and EZH2 are elevated and negatively correlated with CDKN2B in CRC samples

**Figure 7:**
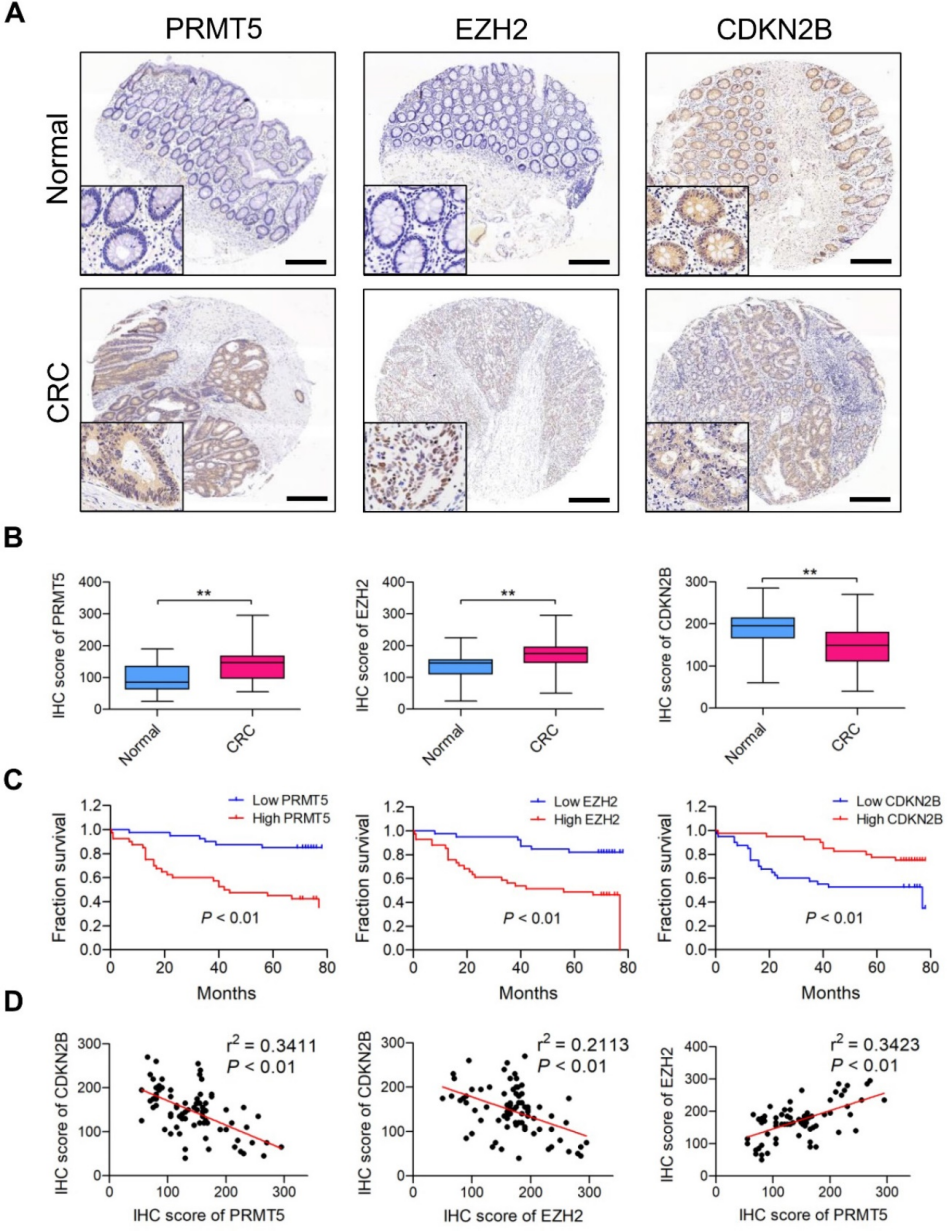
** PRMT5 and EZH2 are elevated and negatively correlated with CDKN2B in CRC samples.** (**A**) Representative images of IHC staining of PRMT5, EZH2 and CDKN2B in 80 cases of CRC patients and their adjacent normal tissues. Scale bar: 200 µm. (**B**) H score of PRMT5 (left), EZH2 (middle) and CDKN2B (right) in CRC (n = 80) and adjacent normal (n = 80) tissues, **P < 0.01. (**C**) Overall survival of patients with CRC was performed by Kaplan-Meier analysis. High and low PRMT5 (left), high and low EZH2 (middle) and high and low CDKN2B (right). *P* < 0.01. (**D**) Correlations of CDKN2B and PRMT5 (left; r^2^ = 0.3411, *P* < 0.01), CDKN2B and EZH2 (middle; r^2^ = 0.2113, *P* < 0.01) and EZH2 and PRMT5 (right; r^2^ = 0.3423, *P* < 0.01) protein levels in CRC tissues (n =80) were performed by Spearman's correlation analysis.

We retrieved Oncomine database and found PRMT5 (4 upregulated and 0 downregulated datasets) and EZH2 (12 upregulated and 0 downregulated datasets) differentially expressed which are upregulated in CRC tissues (*vs*. normal tissues) in different cohorts of datasets (Figure S13); this clue reminds us that PRMT5 and EZH2 might exert oncogenic roles in CRC. Meanwhile, we also screened the Oncomine database and found that CDKN2B (0 upregulated and 20 downregulated datasets) is frequently downregulated in different cohorts of CRC datasets. Furthermore, PRMT5, EZH2 and CDKN2B protein expression levels in CRC samples (80 tumor and 80 normal tissues) were analyzed by IHC staining. As shown in Figure 7A, the majority of CRC biopsies displayed PRMT5- and CDKN2B-positive staining in both cytoplasmic and nuclei of tumor cells, whereas EZH2 was apparently expressed in the nuclei of tumor cells. H score analysis clearly showed that the expression levels of both PRMT5 and EZH2 in CRC tissues were significantly higher than that in adjacent normal tissues, whereas CDKN2B protein was remarkably down-regulated in CRC tissues (Figure 7B). Moreover, the relationship between positive PRMT5/EZH2 expression and clinicopathological parameters, including gender, age, tumor size, tumor stage, lymph node status and distant metastasis, was summarized in Table S4. The higher PRMT5 expression was correlated with tumor size (*P* = 0.0457), tumor stage (*P* = 0.0172) and distant metastasis (*P* = 0.0381). The higher EZH2 expression was correlated with tumor size (*P* = 0.0337) and tumor stage (*P* = 0.0187). Survival analysis suggested that the elevated expression levels of PRMT5 and EZH2 were notably associated with worse outcome of patients with CRC through Kaplan-Meier analysis (Figure 7C; *P* < 0.01). Nevertheless, low CDKN2B expression was correlated with poor overall survival (*P* < 0.01), implying a role as tumor suppressor in CRC. In addition, Pearson's correlation analysis was performed to validate the regulatory effect of PRMT5 and EZH2 on CDKN2B expression in CRC samples. As presented in Figure 7D, PRMT5 (r^2^ = 0.3411, *P* < 0.01) and EZH2 (r^2^ = 0.2113, *P* < 0.01) were negatively correlated with CDKN2B expression in CRC samples, respectively. Meanwhile, PRMT5 was positively correlated with EZH2 (r^2^ = 0.3423, *P* < 0.01). All these findings suggested that PRMT5 and EZH2 upregulation were partly responsible for the reduced CDKN2B expression in CRC.

## Discussion

PRMT5, potentially via H4R3me2s and H3R8me2s deposition at gene promoters, leads to transcriptional repression of pivotal tumor suppressor genes and has been extensively characterized as an oncogene in human malignancies 6. In the current study, our data reveal that depletion of PRMT5 retards CRC cell proliferation and colony formation *in vitro*. Elevated expression of PRMT5 was significantly correlated with poor survival outcome of patients with CRC, indicating that PRMT5 contributes to the progression of CRC. In CRC, transcription factor E2F-1 is di-methylated by PRMT5 and silencing PRMT5 results in elevated E2F-1 protein levels, which coincides with reduced growth rate and apoptosis 33. The disruption of the interaction between PRMT5 and WDR77 significantly inhibited the enzymatic activity of PRMT5 and hence reduced the cell proliferative activity of CRC cells 34. Pak and colleagues suggest that high nuclear expression of PRMT5 is a potentially useful marker for the prediction of submucosal invasion of early CRC 35. Demetriadou and colleagues show that downregulation of PRMT5 leads to activation of RBL2 and CDKN1A (p21^Waf/Cip1^) and repression of EIF4E and FGFR3 in CRC cells 36. Paradoxically, recent studies reveal that PRMT5 strikingly promotes the transcription process of FGFR3 and eIF4E despite depositing repressive histone marks in CRC cells 37, implying that PRMT5 may act upon transcription in a context-dependent manner.

Previous studies established that PRMT5 is associated with ATP-dependent chromatin remodelers (SWI/SNF, NuRD), co-repressors (N-CoR/SMRT, COPR5), developmental modulators (BLIMP1, NF-E4/DNMT3A) and transcription factors/co-activators (p53, MYC, CBP) 7. Notably, one key observation in this study is that EZH2 was identified as a new interacting partner of PRMT5. EZH2, a catalytic component of polycomb repressive complex 2 (PRC2), could specifically deposit a tri-methylation mark at histone H3 lysine 27 (H3K27me3), which is an evolutionarily conserved epigenetic mark associated with transcriptional repression 38. Given the fact that PRMT5-mediated H4R3me2s and H3R8me2s were co-localized with EZH2-mediated H3K27me3 at the core promoter region of CDKN2B gene by ChIP-qPCR assays, it is therefore reasonable to assume that H4R3me2s and H3R8me2s may cross-talk with H3K27me3 histone marks, and lead to a synergic effect on gene repression, but rather at active genes. ChIP-seq profiling using H4R3me2s, H3R8me2s and H3K27me3 antibodies may yield important insights into the cross-talk between H4R3me2s and H3K27me3 or H3R8me2s and H3K27me3 at the whole genome level. Unfortunately, our attempts to detect the H4R3me2s and H3R8me2s deposition peaks in CRC cells by ChIP-seq failed. All currently commercially available H4R3me2s and H3R8me2s antibodies did not efficiently immunoprecipitate H4R3me2s and H3R8me2s histone marks from chromatin. Based on these observations, we speculate that H4R3me2s and H3R8me2s are probably dynamic histone marks and it is therefore hard to form stable immunoprecipitated complexes.

Remarkably, another important finding of this study is that cyclin dependent kinase inhibitor 2B (CDKN2B/p15^INK4b^) was validated as a direct transcriptional target of PRMT5/EZH2 complex in CRC. CDKN2B/p15^INK4b^ is a member of the INK4 family of cyclin-dependent kinase inhibitors, which has been well established as a bona fide tumor suppressor in a variety of human malignancies 39, 40. However, the role of the CDKN2B gene in cancer progression has not been well defined because of CDKN2B inactivation 41. So far, there are a few mechanistic models were proposed to explain the loss of CDKN2B/p15^INK4b^ expression in human diseases, especially in cancers. Staller and colleagues have shown that CDKN2B was repressed by Myc via interacting with Miz-1 in primary mouse embryonic fibroblasts 42. Basu and colleagues revealed that nuclear zinc finger Gfi-1 represses the transcriptional activation of CDKN2B through association with Miz-1 43. Hitomi and colleagues suggested that Oct-1 acts as a transcriptional repressor of the CDKN2B gene during the cellular senescence 44. Furthermore, lncRNAs were associated with epigenetically silencing of CDKN2B, such as SNHG7 45, PVT1 46, FOXC2 47 and BLACAT1 48. It is interesting to note that Jie and colleagues reported that EZH2 substantially impedes CDKN2B activation, which contributes to cellular senescence triggered by EZH2 depletion in gastric cancer cells. Meanwhile, H3K27me3, catalyzed by EZH2, is dramatically detected along the upstream and downstream of the transcription start site of CDKN2B gene by ChIP-qPCR assays 49. However, there are so far no reports that show the links between arginine methylation at histones and the silencing of CDKN2B. Even though a cohort of cell cycle-related genes, including CDKN1A (p21^CIP1/WAF1^), CDKN2C (p18^INK4C^), CDKN1C (p57^KIP2^), p63 and PTEN, were identified as PRMT5 targets in gastric cancer by Liu and colleagues, PRMT5 depletion did not appear to affect the expression of CDKN2B, implying that the mechanisms by which PRMT5 regulates cancer cells in CRC and gastric cancer are different 10. Therefore, illustrating the roles of arginine (R) / lysine (K) methylation at histones and the cross-talk among these histone marks in epigenetically repressing the transcriptional output of CDKN2B in CRC is an importance issue for future studies.

## Conclusion

**Figure 8:**
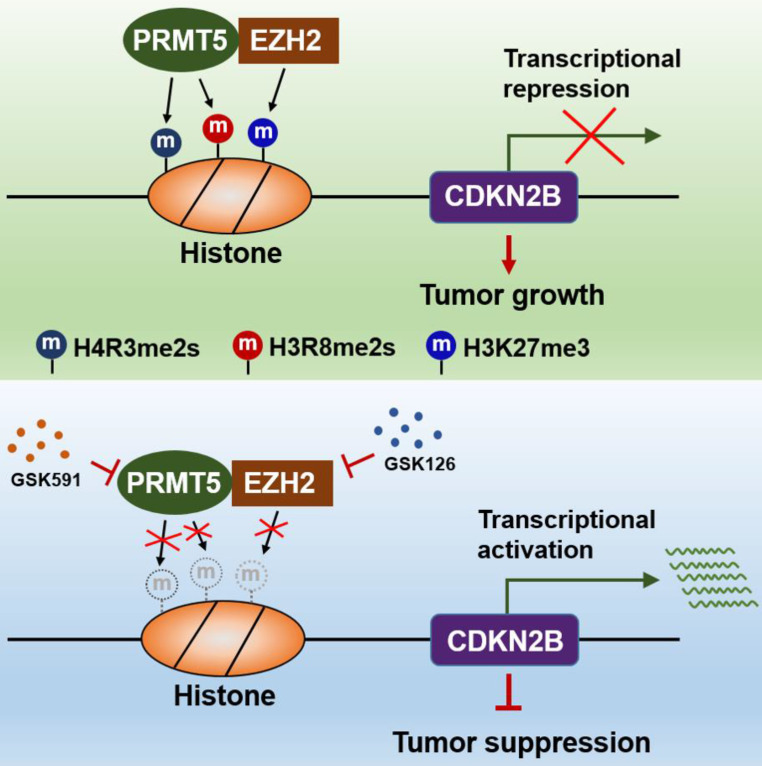
Schematic model of PRMT5/EZH2-mediated epigenetic repression of CDKN2B.

The findings of our work, thereby, reveal that the PRMT5/EZH2/CDKN2B (p15^INK4b^) axis plays a pivotal role in CRC progression. PRMT5 and EZH2 reciprocally interact with one each other and collaborate as a functional unit, providing a new transcriptional regulation model and a new molecular basis for the interplay between histone lysine methylation (H3K27me3) and arginine methylation (H4R3me2s and H3R8me2s) in epigenetically repressing CDKN2B (p15^INK4b^) expression (Figure 8). Although we report a newly identified functional interaction between PRMT5 and EZH2, further investigations are required to understand the biological significance of this association. We also show that combined utilization with PRMT5i (GSK591) and EZH2i (GSK126) to target PRMT5 and EZH2 synergistically attenuates CRC cell growth *in vitro* and* in vivo*. Altogether, our findings not only reveal important insights that link arginine methylation and lysine methylation to directly modulate CDKN2B transcription, but also pave the way to revolutionize our therapeutic options by blocking either PRMT5/EZH2 activities or the associated transcription regulatory network to treat CRC and even probably other cancers.

## Supplementary Material

Supplementary figures and tables.

## Abbreviations

CRC: colorectal cancer
PRMT5: protein arginine methyltransferase 5
EZH2: enhancer of zeste homolog 2
SKB1: Shk1 kinase-binding protein 1
CDKN2B: cyclin dependent kinase inhibitor 2B
PRC2: polycomb repressive complex 2
SAM: S-adenosylmethionine
H3K27me3: tri-methylation of histone H3 at lysine 27
H4R3me2s: symmetric di-methylation of histone H4 at arginine 3
H3R8me2s: symmetric di-methylation of histone H3 at arginine 8
PTM: post-translational modification
MMA: ω-N^G^-monomethylarginine
ADMA: ω-N^G^, N^G^-asymmetric dimethylarginine
SDMA: ω-N^G^, N^G^-symmetric dimethylarginine
FBS: fetal bovine serum
shRNA: short hairpin RNA
Co-IP: co-immunoprecipitation
IHC: immunohistochemical
ChIP: chromatin immunoprecipitation
SDS-PAGE: sodium dodecyl sulfate-polyacrylamide gel electrophoresis
EdU: 5-ethynyl-2'-deoxyuridine
PVDF: polyvinylidene fluoride
HRP: horseradish peroxidase
GST: glutathione s-transferase
IPTG: isopropyl-beta-D-thiogalactopyranoside
PBS: phosphate-buffered saline
HBS: HEPES buffered saline
DAPI: 4, 6-diamidino-2-phenylindole dihydrochloride
H_2_O_2_: hydrogen peroxide
DAB: 3, 3'-diaminobenzidine
CI: combination index
TGI: tumor growth inhibition
