# Supplementary material for: PRMT5 functionally associates with EZH2 to promote colorectal cancer progression through epigenetically repressing CDKN2B expression
Source: Theranostics. 2021 Jan 27;11(8):3742–59. doi: 10.7150/thno.53023 (PMC7914347; doi:10.7150/thno.53023)
Supplement: Supplementary file 1 — Supplementary figures and tables. [file thnov11p3742s1.pdf]

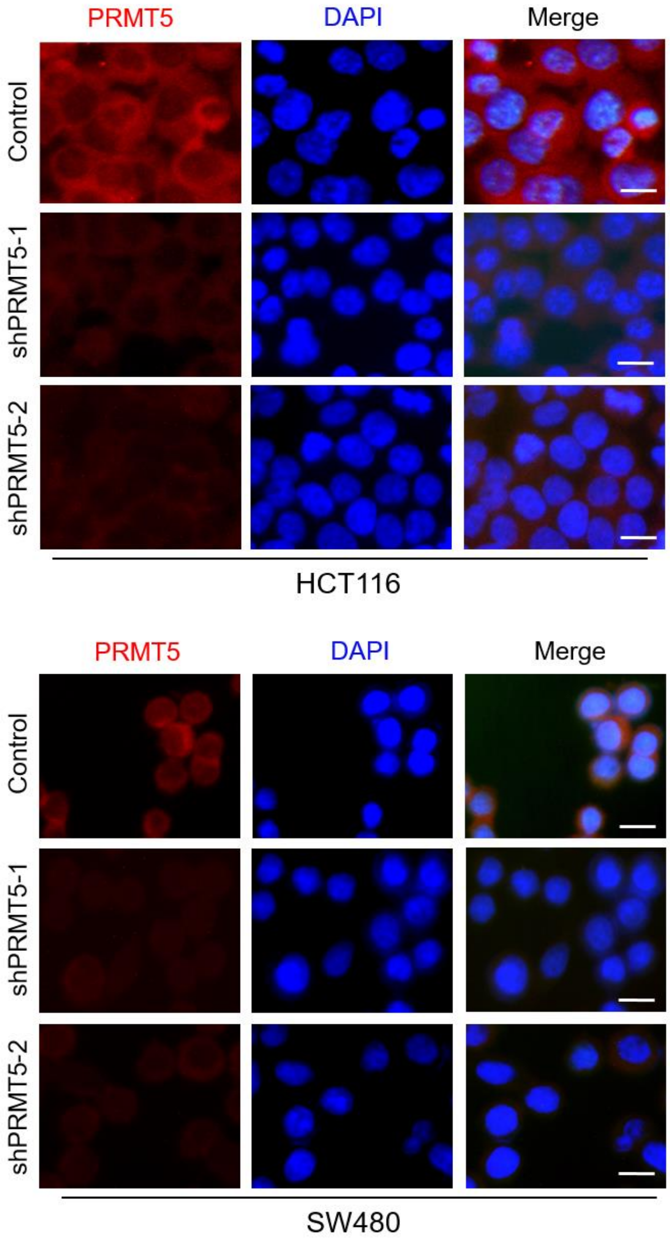

Figure S1. The knockdown of PRMT5 was verified by immunofluorescence microscopy in Control, shPRMT5-1 and shPRMT5-2-treated HCT116 and SW480 cells. Scale bar: 10 μm.

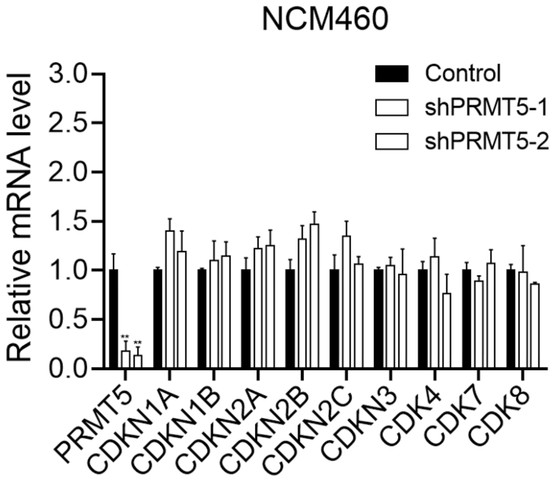

Figure S2. Relative mRNA expression of genes involved in the regulation of cell proliferation and cell cycle was determined by real-time PCR in shPRMT5-1 and shPRMT5-2-infected NCM460 cells compared with control cells. GAPDH was used as an internal control.

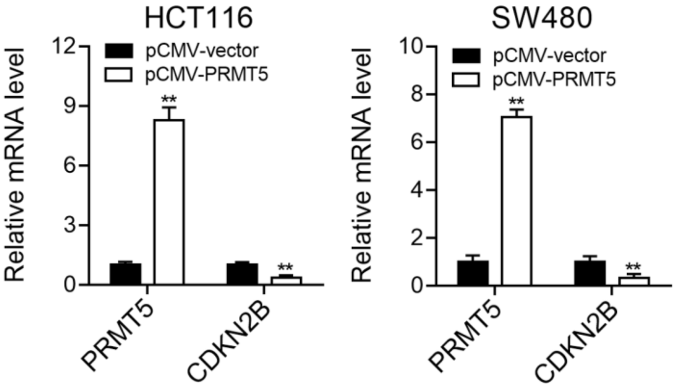

Figure S3. Relative mRNA expression of CDKN2B was determined by real-time PCR in PRMT5-overexpressed HCT116 and SW480 cells compared with control cells. GAPDH was used as an internal control. $**P < 0.01$.

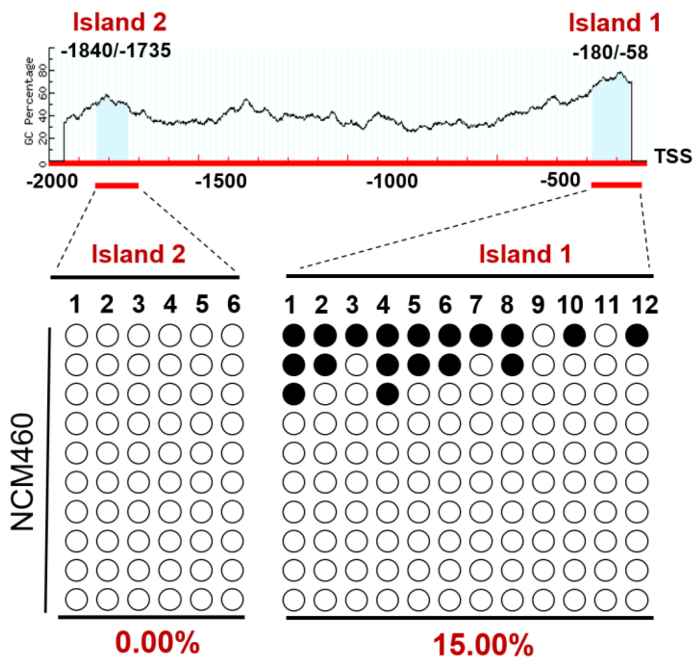

Figure S4. Bisulfite sequencing analysis of the CDKN2B promoter (CpG island 1: -1840 ~ -1735, CpG island 2: -180 ~ -58) in noncancerous NCM460 colon cells.

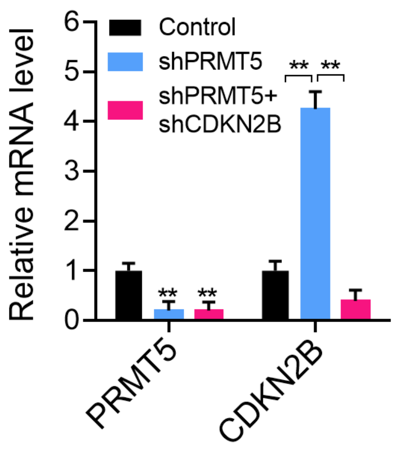

Figure S5. Relative mRNA expression levels of PRMT5 and CDKN2B were determined by real-time PCR assays in Control, shPRMT5 and shPRMT5 + shCDKN2B-treated HCT116 xenograft tumors excised from mice. ***P* < 0.01.

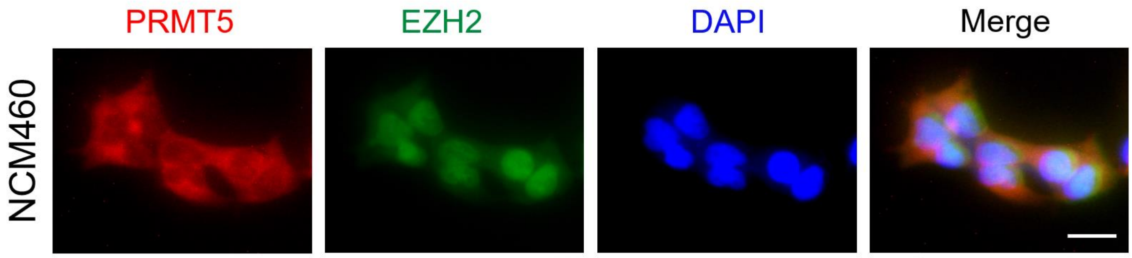

Figure S6. The subcellular location of endogenous PRMT5 and EZH2 proteins was analyzed in noncancerous NCM460 colon cells by immunofluorescence microscopy. Scale bar: 10 μm.

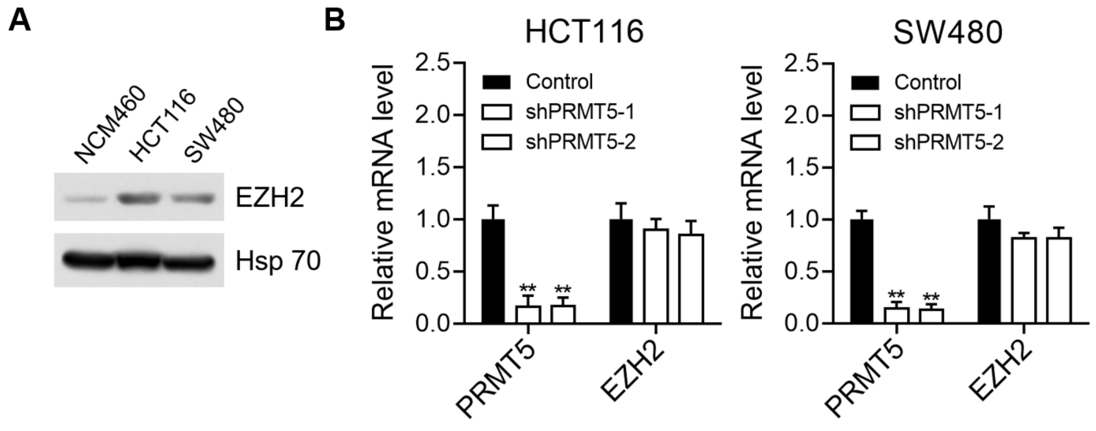

Figure S7. (**A**) Relative protein expression of EZH2 was determined by Western blot in NCM460, HCT116 and SW480 cells. Hsp 70 was used as an internal control. (**B**) Relative mRNA expression of EZH2 was determined by real-time PCR in HCT116 and SW480 cells stably expressing control shRNA (negative control), shPRMT5-1 and shPRMT5-2. GAPDH was used as an internal control. **$P < 0.01$.

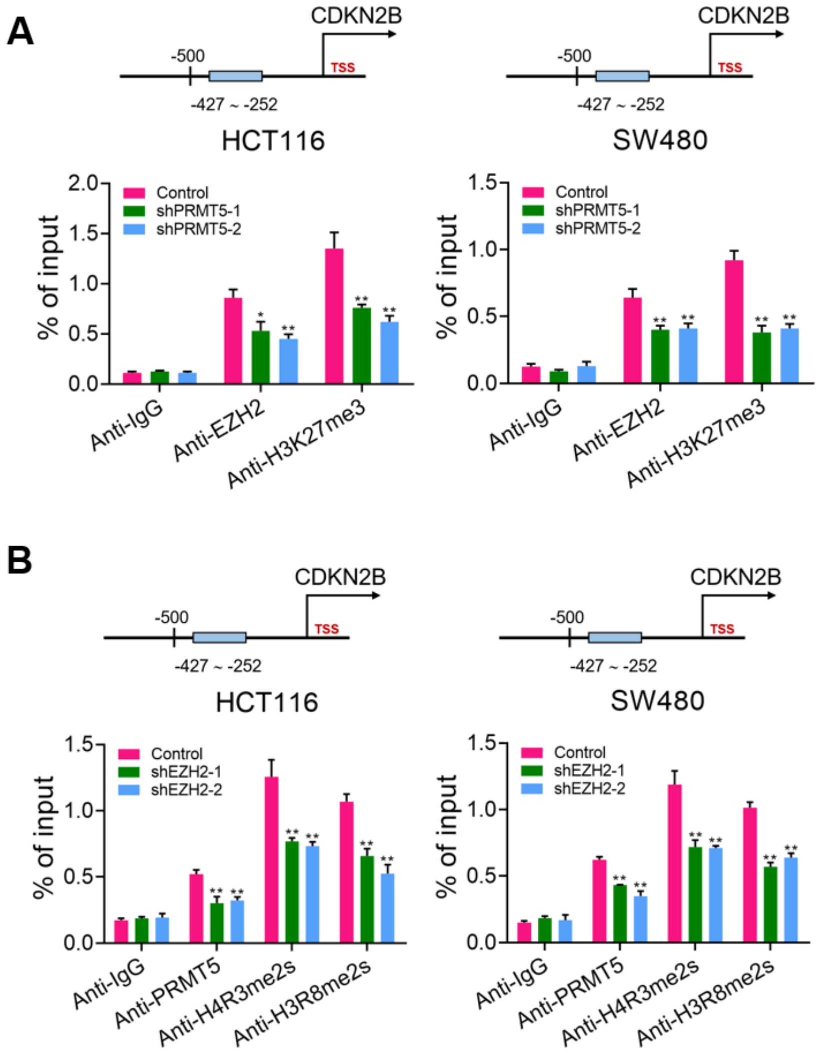

Figure S8. (**A**) Relative enrichment of EZH2 and its catalytic histone mark H3K27me3 on the promoter region of CDKN2B gene (Primer 2: -427 ~ -252) was evaluated by ChIP-qPCR assays in PRMT5-depleted HCT116 and SW480 cells. IgG was used as a negative control. **P* < 0.05, ***P* < 0.01. (**B**) Relative enrichment of PRMT5 and its catalytic histone marks H4R3me2s and H3R8me2s on the promoter region of CDKN2B gene (Primer 2: -427 ~ -252) was evaluated by ChIP-qPCR assays in EZH2-depleted HCT116 and SW480 cells. IgG was used as a negative control. ***P* < 0.01.

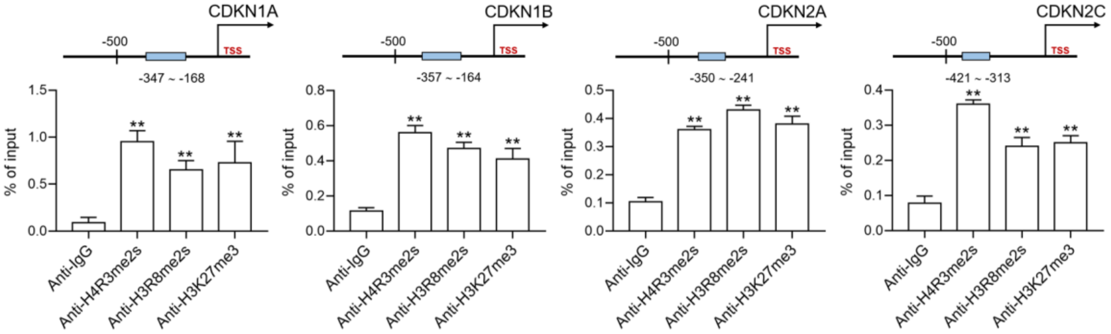

Figure S9. Relative enrichments of H4R3me2s, H3R8me2s and H3K27me3 on the promoter regions of CDKN1A (Primer: -347 ~ -168), CDKN1B (Primer: -357 ~ -164), CDKN2A (Primer: -350 ~ -241) and CDKN2C (Primer: -421 ~ -313) were evaluated by ChIP-qPCR assays in HCT116 cells. IgG was used as a negative control. $^{**}P < 0.01$.

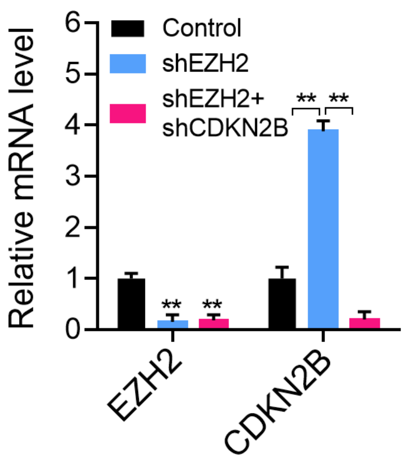

Figure S10. Relative mRNA expression levels of EZH2 and CDKN2B were determined by real-time PCR assays in Control, shEZH2 and shEZH2 + shCDKN2B-treated HCT116 xenograft tumors excised from mice. **$P < 0.01$.

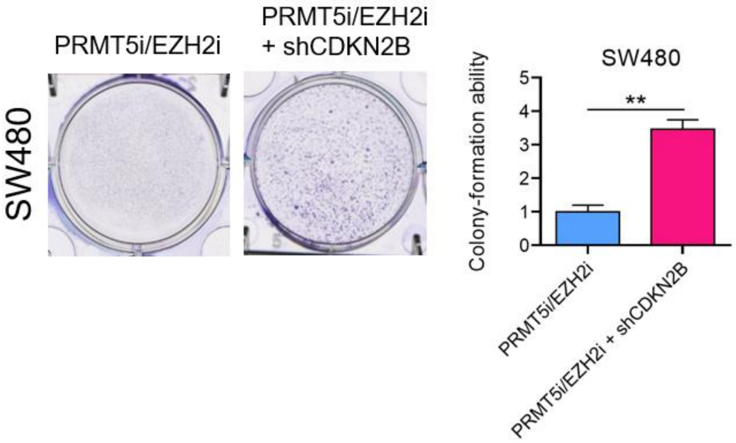

Figure S11. Colony formation capability of SW480 cells treated with PRMT5i/EZH2i and PRMT5i/EZH2i + shCDKN2B by plate colony-formation assays. Quantifications are shown on the right panel. $**P < 0.01$.

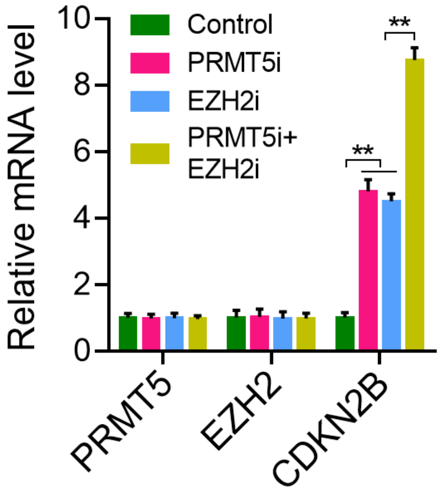

Figure S12. Relative mRNA expression levels of PRMT5, EZH2 and CDKN2B were determined by real-time PCR assays in Control, PRMT5i (GSK591), EZH2i (GSK126), or PRMT5i (GSK591) + EZH2i (GSK126)-treated HCT116 xenografts; n = 7, $^{**}P < 0.01$.

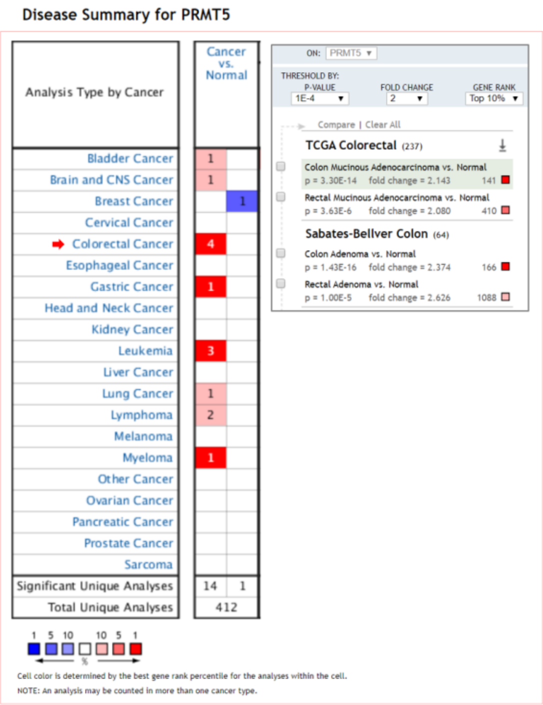

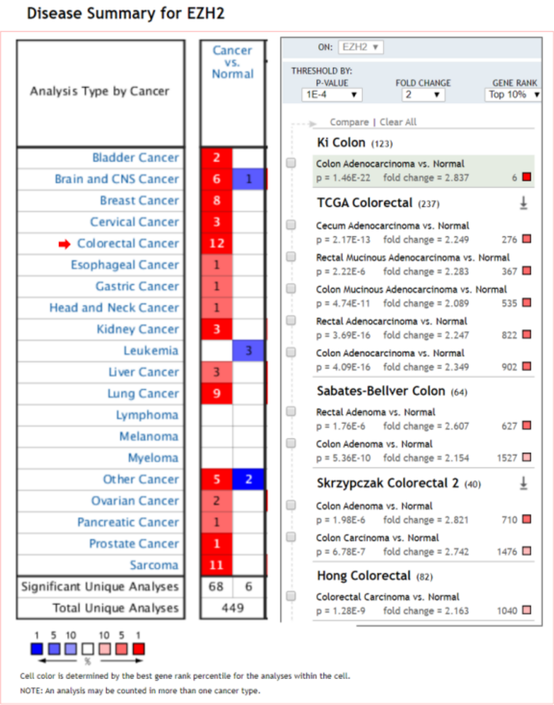

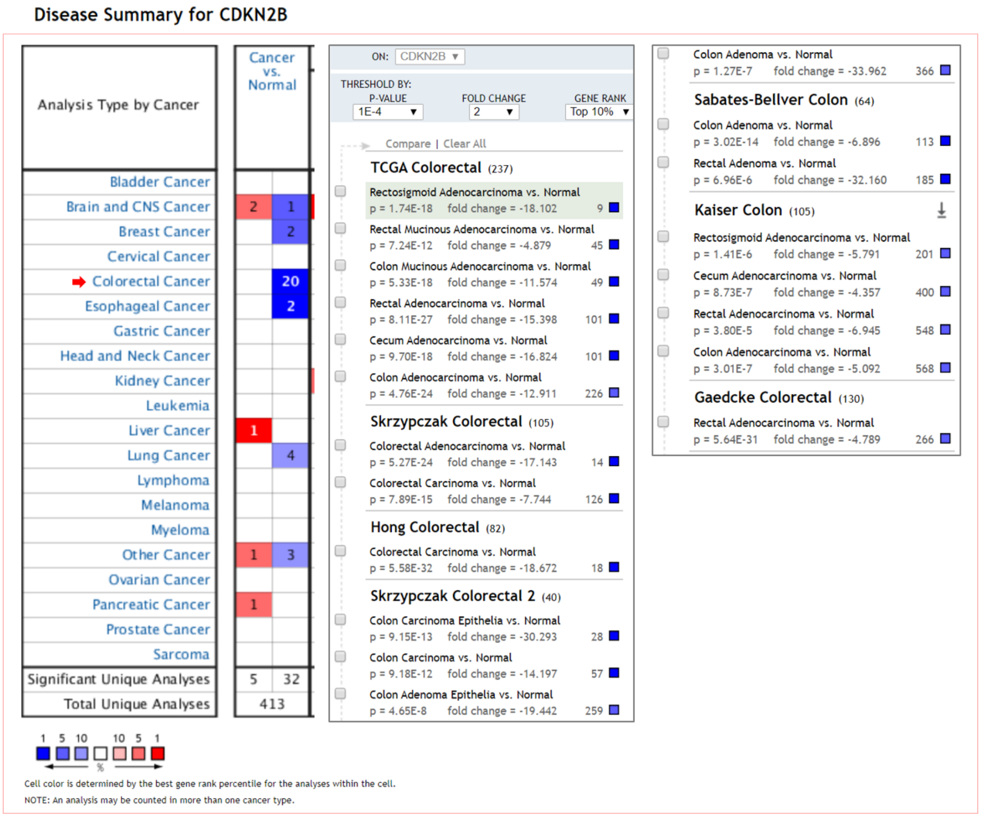

Figure S13. Differential expressions of PRMT5, EZH2 and CDKN2B in different tumors. The available data was assessed using the Oncomine database (https://www.oncomine.org) according to the following criteria: (I) gene: PRMT5,

EZH2 and CDKN2B; (II) cancer type: colorectal cancer; (III) data type: all; (IV) analysis type: cancer analysis vs. normal analysis; (V) thresholds: *P* value <1E−4, fold change >2 and gene rank = top 10%.

Table S1. The primer sequences used for real-time PCR analysis

**Gene name**	**Sequence** (5'-3')
GAPDH forward	5'-ATGACCCCTTCATTGACCTCA-3'
GAPDH reverse	5'-GAGATGATGACCCTTTTGGCT-3'
PRMT5 forward	5'-CTGTCTTCCATCCGCGTTTCA-3'
PRMT5 reverse	5'-GCAGTAGGTCTGATCGTGTCTG-3'
EZH2 forward	5'-CGCTTTTCTGTAGGCGATGT-3'
EZH2 reverse	5'-TGGGTGTTGCATGAAAAGAA-3'
CDKN1A forward	5'-CGATGGAACTTCGACTTTGTCA-3'
CDKN1A reverse	5'-GCACAAGGGTACAAGACAGTG-3'
CDKN1B forward	5'-AATTGGGGCTCCGGCTAACT-3'
CDKN1B reverse	5'-TGCAGGTCGCTTCCTTATTCC-3'
CDKN2A forward	5'-GTGGGTTTGTAGAAGCAGGC-3'
CDKN2A reverse	5'-ATCGGGGATGTAATGCCAGG-3'
CDKN3 forward	5'-TCCGGGGCAATACAGACCAT-3'
CDKN3 reverse	5'-GCAGCTAATTTGTCCCGAAACTC-3'
CDK4 forward	5'-ATGGCTACCTCTCGATATGAGC-3'
CDK4 reverse	5'-CATTGGGGACTCTCACACTCT-3'
CDK7 forward	5'-TGTATGGTGTAGGTGTGGACA-3'
CDK7 reverse	5'-TGCAAAGGTATTCCAGGGAAAC-3'
CDK8 forward	5'-ACCTGTTTGAATACGAGGGCT-3'
CDK8 reverse	5'-TGCCGACATAGAGATCCCAGT-3'

Table S2. The sequences of the primers used for bisulfite sequencing

Name	Sequence (5’-3’)
Island 1 forward	5’-GCCCAGTCTCTGGCGCATGCGTCCT-3’
Island 1 reverse	5’-CCACCCCCTTAGGCTCCGCCCCCTG-3’
Island 2 forward	5’-GGACCACCCAGGCCTGAGTATAAGC-3’
Island 2 reverse	5’-GTTGACTGAATGAAAAACAACTTTG-3’

Table S3. The sequences of the primers used for ChIP-qPCR

**Name**	**Sequence (5’-3’)**
CDKN2B Primer 1 forward	5’-AGAAGGACGACGGGAGGGTAATG-3’
CDKN2B Primer 1 reverse	5’-GATAATCCACCGTTGGCCGTAAA-3’
CDKN2B Primer 2 forward	5’-TGCAGAGCTGTCGCTTTCA-3’
CDKN2B Primer 2 reverse	5’-AATGTTCACCACTGCCCTCA-3’
CDKN2B Primer 3 forward	5’-AATGACAAGCCCAGCACCA-3’
CDKN2B Primer 3 reverse	5’-AATCTGAGATCCCAGTTTC-3’
CDKN2B Primer 4 forward	5’-GGGGTTGGATGGGAAAGAA-3’
CDKN2B Primer 4 reverse	5’-TGCTGGTTAGGGCTGAAAA-3’
CDKN2B Primer 5 forward	5’-CTTTGAAGTCTGGTAAGGGTG-3’
CDKN2B Primer 5 reverse	5’-ATCATTGTTGGAGGTGGGT-3’
CDKN1A Primer forward	5’-GCTGCATTGGGTAAATCCTTG-3’
CDKN1A Primer reverse	5’-AGTCCCTCGCCTGCGTTGGT-3’
CDKN1B Primer forward	5’-GGAGGGAGGTCGGGGCTTAG-3’
CDKN1B Primer reverse	5’-CGGGAGATTGGCTGGTCGC-3’
CDKN2A Primer forward	5’-TACAGGTGATTTCGATTCTCGG-3’
CDKN2A Primer reverse	5’-GGGTGTTTGGTGTCATAGGGA-3’
CDKN2C Primer forward	5’-CTGAGGAACGACTCCCTTTATGCC-3’
CDKN2C Primer reverse	5’-GGGTTTGTTATTTAAGACGGTTGTGG-3’

Table S4. Clinicopathologic characteristics of PRMT5 and EZH2 expression in CRC patients

Characteristics	Cases	H score of PRMT5 (mean ± SD)	*P* value*	H score of EZH2 (mean ± SD)	*P* value*
Gender					
Male	41	144.3±54.4	0.4786	172.2±49.1	0.4016
Female	39	143.7±56.5		169.2±58.0	
Age					
>65	41	141.8±52.0	0.3566	170.0±53.8	0.4473
≤65	39	146.4±58.8		171.6±53.6	
Tumor size					
≥5 cm	45	153.6±59.6	0.0457	180.5±51.0	0.0337
<5 cm	35	131.8±47.4		158.3±54.4	
Tumor stage					
I-II	66	138.0±48.9	0.0172	165.0±51.2	0.0187
III-IV	14	172.5±72.7		197.7±56.7	
Lymph node status					
N0	59	141.0±53.0	0.2069	167.0±51.6	0.1463
N1-3	21	152.6±60.9		181.5±57.7	
Distant metastasis					
M0	3	141.8±54.4	0.0381	170.0±53.9	0.2489
M1	77	200.0±52.1		191.7±41.9	

**P* values were determined by Student's *t* test to compare values for the two parameters in each category.
